# Ca^2+^ Influx and Tyrosine Kinases Trigger *Bordetella* Adenylate Cyclase Toxin (ACT) Endocytosis. Cell Physiology and Expression of the CD11b/CD18 Integrin Major Determinants of the Entry Route

**DOI:** 10.1371/journal.pone.0074248

**Published:** 2013-09-13

**Authors:** Kepa B. Uribe, César Martín, Aitor Etxebarria, David González-Bullón, Geraxane Gómez-Bilbao, Helena Ostolaza

**Affiliations:** Unidad de Biofísica (CSIC, UPV/EHU), and Departamento de Bioquímica, Universidad del País Vasco, UPV/EHU, Bilbao, Spain; Institut Curie, France

## Abstract

Humans infected with *Bordetella pertussis*, the whooping cough bacterium, show evidences of impaired host defenses. This pathogenic bacterium produces a unique adenylate cyclase toxin (ACT) which enters human phagocytes and catalyzes the unregulated formation of cAMP, hampering important bactericidal functions of these immune cells that eventually cause cell death by apoptosis and/or necrosis. Additionally, ACT permeabilizes cells through pore formation in the target cell membrane. Recently, we demonstrated that ACT is internalised into macrophages together with other membrane components, such as the integrin CD11b/CD18 (CR3), its receptor in these immune cells, and GM1. The goal of this study was to determine whether ACT uptake is restricted to receptor-bearing macrophages or on the contrary may also take place into cells devoid of receptor and gain more insights on the signalling involved. Here, we show that ACT is rapidly eliminated from the cell membrane of either CR3-positive as negative cells, though through different entry routes, which depends in part, on the target cell physiology and characteristics. ACT-induced Ca^2+^ influx and activation of non-receptor Tyr kinases into the target cell appear to be common master denominators in the different endocytic strategies activated by this toxin. Very importantly, we show that, upon incubation with ACT, target cells are capable of repairing the cell membrane, which suggests the mounting of an anti-toxin cell repair-response, very likely involving the toxin elimination from the cell surface.

## Introduction

Adenylate cyclase toxin (ACT), a ≈ 200 kDa protein, is an essential virulence factor secreted by *Bordetella pertussis* [1], the whooping cough bacterium. Whooping cough or pertussis is a severe infant respiratory disease that is deadly again in spite of the high vaccine coverage, due to the worldwide resurgence of more virulent forms of the bacterium [2]. ACT is a member of the calcium-binding, pore-forming RTX (Repeats-in-Toxin) family of protein toxins that share a characteristic calcium-binding motif of Gly- and Asp-rich nonapeptide repeats and a marked cytotoxic and cytolytic activity [3]–[5]. ACT exhibits besides a distinctive feature: it has an amino-terminal adenylate cyclase catalytic domain (AC domain). The RTX component forms pores, apparently oligomeric, in cell membranes and serves as the cell binding domain [6], [7]. The AC domain converts ATP into cAMP in a reaction that is stimulated by eukaryotic calmodulin [8]. To generate cAMP, ACT binds to cells, translocates the AC domain directly across the plasma membrane [9] and catalyzes the unregulated conversion of intracellular ATP to cAMP.

ACT exhibits multiple effects, both cAMP-dependent and independent. ACT generated cAMP inhibits bactericidal functions of neutrophils and macrophages such as generation of oxidative burst and killing of phagocytized bacteria [10], and induces apoptotic cell death [11], cell cycle arrest [12] and dysregulation of the adaptative immune system [13]–[15]. The pore-forming activity of ACT has been involved in IL-1β production by dendritic cells through activation of caspase-1 and the NALP3-containing inflammasome complex [16] and, together with cAMP accumulation and cellular ATP depletion, pore formation by ACT synergistically contributes to non-apoptotic cell death [12], [17]. ACT induces influxes of calcium ions, raising the intracellular concentration of this cation [18], [19].

The β2 integrin CD11b/CD18 (α_M_β_2_, CR3 or Mac-1) has been demonstrated to enhance the sensitivity of cells to intoxication by ACT [20], [21], however, cells that do not express the integrin are as well effectively intoxicated by this toxin [22], [23], suggesting that interaction with a specific receptor is not an absolute requirement for ACT to affect target cells. ACT is able to associate with artificial pure lipid membranes, produce ion-conducting channels and elicit marker release from liposomes containing no protein [24], [25].

In a recent work our group has demonstrated that ACT is internalised into macrophages together with other membrane components, such as the integrin CD11b/CD18 and GM1. The toxin-triggered internalisation occurs in parallel through two different routes of entry, chlorpromazine-sensitive receptor-mediated endocytosis and clathrin-independent internalisation [26]. An intracellular vesicular localization of ACT has also been observed in T cells by other authors [15].

Previous studies have documented the capacity of nucleated mammalian cells to cope with a limited number of membrane lesions caused by pore-forming toxins, and recover, through a membrane-repair response which involves the endocytosis of the toxin from the membrane [27]–[30]. Here we sought to determine whether ACT uptake may take place also into cells devoid of receptor and discern whether the toxin is able to trigger signalling events, independently of its association with the integrin, which could activate endocytic events into the cells, and whether this uptake might be related with cell repair responses. Performing a detailed analysis addressing the endocytosis of ACT in several cell types: CHO-K1 cells, CR3^+^CHO-K1 cells, and CR3**^−^**J774A.1 cells, we find that ACT is indeed rapidly removed from the cell membrane of cells either CR3-positive as negative, though through different entry pathways depending on the cell type. ACT-induced Ca^2+^ influx and activation of Tyr kinases are required for the different endocytic strategies activated by this toxin. In the case of the CHO-K1 cells, phosphorylation of caveolin is also involved in the internalisation of ACT. Remarkably, we show that cells injured by low ACT doses are able to restore their membrane, very likely through toxin elimination from the cell membrane suggesting that a repair response might be triggered in the target cell to protect it from the toxin insult.

## Experimental Procedures

### Antibodies and Reagents

Anti-RTX, clathrin HC siRNA, caveolin-1 siRNA and control siRNA were from Santa Cruz Biotechnology (Santa Cruz, CA, USA); anti-caveolin-1, Cytochalasin D, methyl-β-cyclodextrin, nocodazole, nystatin, filipin, nifedipine, chlorpromazine, DMA, and genistein from Sigma-Aldrich (St Louis, MI, USA); anti-CD11b, anti-GAPDH, anti-P-caveolin-1 and anti-clathrin were from abcam (Cambridge, UK); KT5620, PP2 and PP3 were from Calbiochem (Merck, Germany); anti-mouse Texas Red, anti-rabbit FITC and BODIPY® FL C5 lactosylceramide complexed to BSA, from Invitrogen, Molecular Probes (Carlsbad, CA, USA). FITC-labeled transferrin was from Exbio (Praha, Czech Republic).

### ACT Purification

ACT was expressed in *Escherichia coli* XL-1 blue cells (Stratagene) transformed with pT7CACT1 plasmid, kindly provided by Dr. Peter Sebo (Institute of Microbiology of the ASCR, v.v.i., Prague, Czech Republic) and purified as previously described [25].

### Cell Culture

CHO-K1 cells (ATTC, number CCL-61) were cultured at 37°C in DMEM supplemented with 10% (v/v) FBS, and 4 mM L-glutamine in a humidified atmosphere with 5% CO_2_. CR3^+^CHO-K1 cells (Innoprot, Spain) were cultured in DMEM supplemented with 10% (v/v) FBS, 150 µg/ml hygromycin and 4 mM L-glutamine. J774A.1 macrophages (ATTC, number TIB-67) were grown at 37°C in DMEM containing 10% (v/v) FBS, and 4 mM L-glutamine in a humidified atmosphere with 5% CO_2._

### CD11b/CD18 Stably Knocked-down Cell Line: CR3^−^ J774A.1

J774A.1 cell line was stably transfected with a pool of shRNA targeted against CD11b subunit, thereby obtaining a derived cell line that does not express the CR3. Integrin knockdown was achieved according the protocol supplied by manufacturer (Santa Cruz Biotechnology, CA, USA). Briefly, cells were grown in 6-well tissue culture plates until 50–70% of confluence in antibiotic-free normal growth medium, washed twice with OptiMEM (Invitrogen, USA) and transfected with a shRNA Plasmid DNA:shRNA Plasmid Transfection Reagent ratio of 1 µg : 3 µL. After 48 h of transfection, stably transfected cells were selected by 1.5 µg/mL puromycin and grown in normal culture medium supplemented with puromycin. The same protocol was followed to obtain the J774A.1-puro+ cells, a derived cell line transfected with a control shRNA. As show in **Figure S1** the expression of CR3 in the knocked down cell line was almost negligible.

### Measurement of ACT Internalisation by FACS

Internalisation kinetics of ACT, in CHO-K1 and CR3**^−^**J774A.1, or of ACT and integrin in J774A.1 and CR3^+^CHO-K1 cell lines was determined by flow cytometry as previously described [26]. Briefly, macrophages were incubated with 35 nM ACT for 2 min at 37°C to allow an irreversible binding of toxin to the cells. The rationale of such incubation time is that the initial events leading to toxin internalisation have not yet been triggered. Then, cells were washed 3 times with ice-cold PBS to remove unbound toxin, and incubated again at 37°C for various intervals (0–30 min). Cells were then washed and detached in ice-cold PBS, labeled under non-permeabilizing conditions against β2 integrins or ACT with the appropriate antibodies for 1 h at room temperature, then centrifuged, washed and mixed in buffer containing FITC-conjugated secondary antibodies and incubated for 1 h at room temperature. Then, cells were washed, resuspended in ice-cold PBS and analyzed in a FACSCalibur flow cytometer (Beckton Dickinson). Geometric mean fluorescence intensities (GMFI) were used to show ACT internalisation kinetics in CHO-K1 cells. When inhibitors were used and to better illustrate their inhibitory effect, the percentage of internalisation at each point was calculated as follows: 100– (percentage of GMFI of the cells at each point of the kinetic relative to the GMFI of the cells obtained after toxin binding for 2 min).

### Analysis of Transferrin or Lactosylceramide Uptake

Cells were incubated with serum free medium for 1 h at 37°C. When methyl-β-cyclodextrin (10 mM), filipin (6 µg/ml) or nystatin (25 µg/ml) were used, the cells were incubated with these inhibitors during 30 minutes at 37°C. Then, cells were incubated with FITC-labeled transferrin or BODIPY-labeled lactosylceramide complexed to BSA during 10 minutes at 4°C to allow binding and then challenged to 37°C and incubated for 10 minutes with or without 35 nM ACT to follow the endocytosis. After treatment, cells were washed in ice-cold PBS and fixed with 3.7% paraformaldehyde for 10 minutes. The intensity of the internalised fluorescent molecules was measured in a BD FACSCalibur flow cytometer and was used to show the extent of FITC-labeled transferrin or BODIPY-labeled lactosylceramide internalisation.

### Isolation and Analysis of Detergent Resistant Membranes (DRMs)

DRMs from CHO-K1 cells were prepared according to Martín et al. [26]. Briefly, control and 35 nM ACT-treated cells for 5 min at 37°C were lyzed in 10 mM Tris-HCl, 200 mM NaCl, 1 mM EDTA pH 7.4, containing 1% (v/v) Triton X-100, for 30 minutes at 4°C. The extracts were brought to 40% sucrose and placed at the bottom of 5–35% sucrose gradient with 1% TX-100. Gradients were ultracentrifuged for 18 hours at 188,000 g at 4°C. Fractions of 1 ml were harvested from the top of the gradient and stored at −20°C. Equal amounts of protein from each fraction were analysed by SDS-PAGE and Western blot.

### Western Blotting

Proteins were separated electrophoretically on 8.5% SDS-polyacrylamide gels and transferred to nitrocellulose membrane. The membranes were then blocked overnight at 4°C, and after 2 h of incubation with the corresponding primary antibodies, membranes were washed and exposed to the secondary antibodies for 1 h at room temperature. Proteins were detected using the enhanced chemiluminiscence detection system (ECL®, Amersham Biosciences). The Quantity One® Image Analyzer software program (Bio-Rad) was used for quantitative densitometric analysis.

### Confocal Microscopy

Cells were grown to sub-confluency onto 12 mm diameter glass coverslips placed into the wells of a 24-well plate. 35 nM ACT was added to the medium and cells were incubated for 10 min. Then, treated cells were washed three times in PBS to remove unbound toxin, fixed for 10 min with 3.7% paraformaldehyde and permeabilized with acetone at –20°C. Control cells followed the same procedure. Then samples were incubated with the appropriate primary antibodies for 1 h followed by incubation with Texas Red- or FITC-conjugated secondary antibodies. Coverslips were mounted on a glass slide and samples were visualized using a confocal microscope (Olympus IX 81) with sequential excitation and capture image acquisition with a digital camera (Axiocam NRc5, Zeiss). Images were processed with Fluoview v.50 software.

### Measurements of Intracellular [Ca^2+^]

Calcium influx into CR3^+^CHO-K1 and CR3**^−^**J774A.1 cells was measured as previously described [18]. Briefly, cells grown on glass coverslips, were loaded with 2 µM fura2-AM for 30–45 min. The coverslips were mounted on a termostatized perfusion chamber on a Nikon Eclipse TE 300 based microspectofluorometer and visualized with a ×40 oil-immersion fluorescence objective lens. At the indicated time, 35 nM ACT was added and the intracellular Ca^2+^ levels were determined using the method of Grynkiewicz et al. [31]. The ratio of light exited at 340 nm to that at 380 was determined with a Delta-Ram system (Photon Technologies International, Princeton).

### Transfection of CHO-K1 and CR3^+^CHO-K1 Cells with siRNA

50% confluent cells were transfected with 20 nM siRNA against caveolin-1 or clathrin using oligofectamine™ transfection reagent in OPTIMEM medium (Invitrogen) as directed by the manufacturer. 5 h following transfection medium was supplemented with foetal bovine serum, and the incubation continued for 72 h. A scrambled sequence siRNA was used as a negative control. siRNA interfered cells were then incubated with 35 nM ACT for 10 min to determine ACT or integrin internalisation by FACS as described above or for protein expression quantification by Western blotting to determine the extent of protein silencing.

### Membrane Permeabilization Assayed by propidium Iodide Uptake

Cells cultured on coverslips at 80% confluence were treated for different times (5, 10, 30, 60 and 120 min) with 35 or 5 nM ACT at 37°C, washed three times with ice-cold PBS supplemented with 1% BSA, trypsinized, and washed again three times before adding 50 µg/mL PI (Sigma) for 1 min to assess pore formation and membrane repair. PI incorporated into cell was analyzed in a FACSCalibur flow cytometer (Beckton Dickinson). Population of viable cells which show PI staining were gated in a FSC *vs* PI density plot and the ratio of the ACT-permeabilized cells to non permeabilized control cells on the gated cell population was calculated.

### Statistical Analysis

All measurements were performed at least 3 times and levels of significance were determined by a two-tailed Student’s *t*-test.

## Results

### ACT is Internalised in CHO-K1 Cells by a Clathrin-independent Pathway

CHO-K1 cells do not constitutively express the CD11b/CD18 integrin, however its intracellular cAMP level is substantially elevated by ACT at concentrations above 100 ng/ml, with a maximum level of accumulation of the second messenger comparable to that achieved in CR3^+^ cells [22]. The use of CHO-K1 cells as model CR3**^−^** target to characterize ACT activity has been extensive [22].

**Figure 1:**
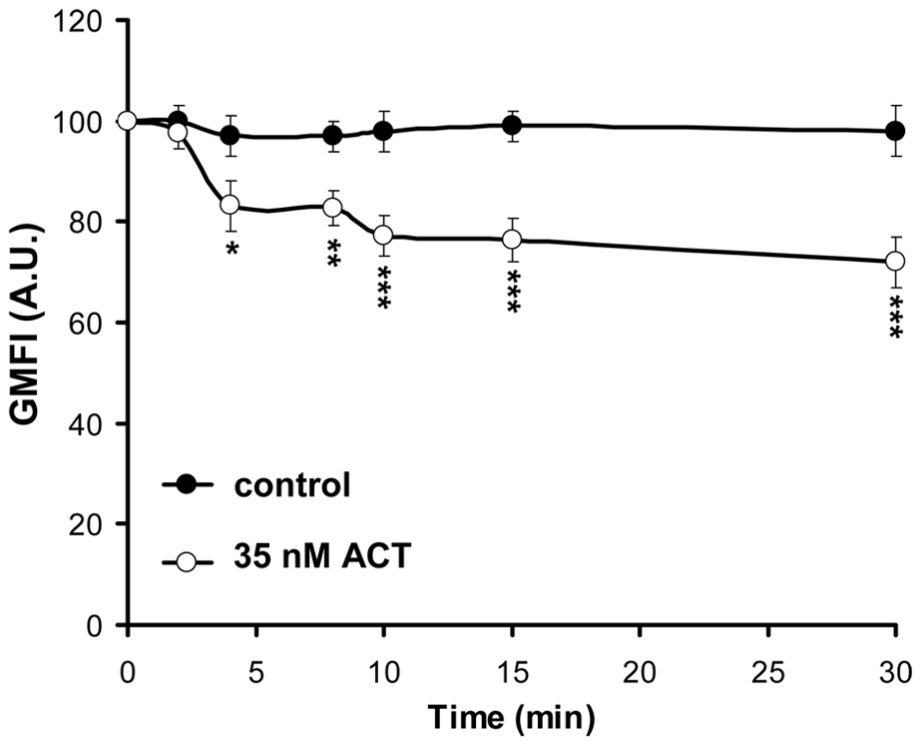
Kinetics of ACT internalisation into CHO-K1 cells. Addition of ACT (35 nM) to CHO-K1 cells results in time-dependent internalisation of the toxin, as determined by flow cytometry and described in *Materials and Methods*. Geometric mean fluorescence intensities (GMFI) were used to show ACT internalisation. The data shown are the mean ± SD of at least three independent experiments, with *p<0.05, **p<0.025 and ***p<0.001.

As determined by flow cytometry, ACT toxin is internalised in a time dependent manner in CHO-K1 cells treated with ACT at 37°C. After 30 min incubation 33±5% of the toxin bound to the cells is internalised (**Fig. 1**).

**Figure 2:**
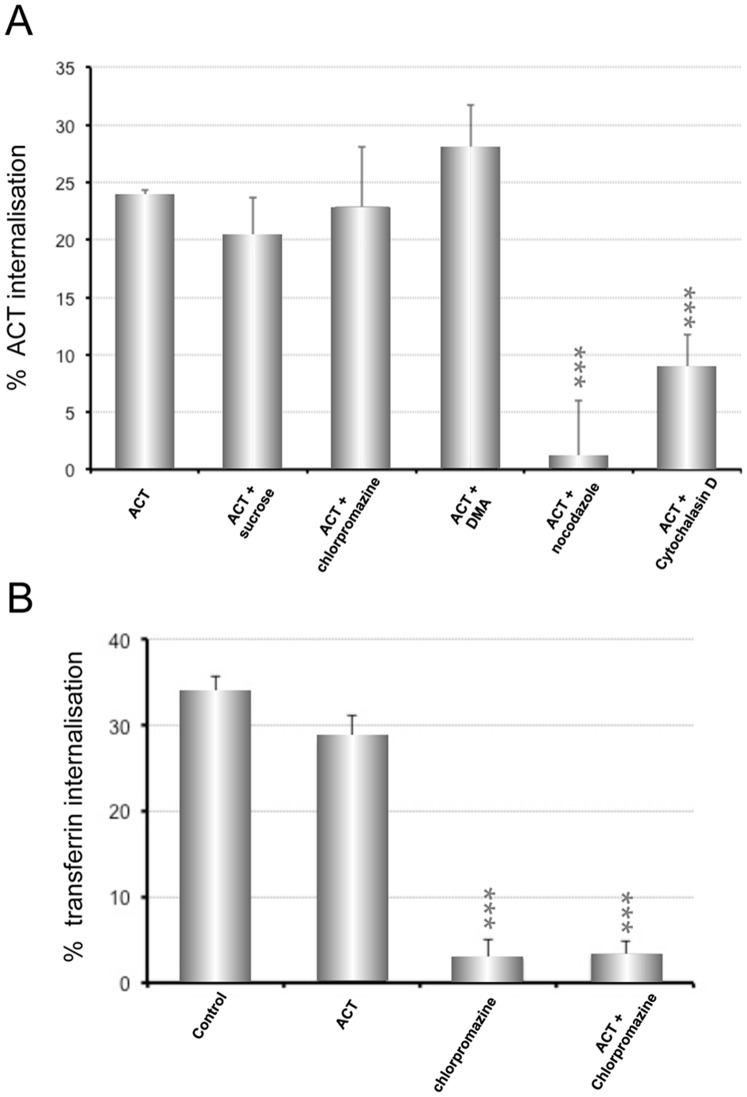
Effect of several inhibitors on the internalisation of ACT into CHO-K1 cells. (**A**) CHO-K1 cells were pre-incubated for 30 min at 37°C with several known inhibitors of endocytosis routes, at the following concentrations: sucrose (450 mM), chlorpromazine (5 µg/ml), DMA (200 µM), nocodazole (20 µM), cytochalasin D (10 µM). Then toxin was added at a final concentration of 35 nM, and cells further incubated at 37°C for 10 min. Then, the surface staining of ACT was measured using a flow cytometer. (**B**) Transferrin uptake by the CHO-K1 cells was used as a control assay to be sure that the clathrin-dependent endocytosis was fully functional, and that ACT did not interfere with such internalisation. Internalisation was assessed by FACS and expressed as described in *Materials and Methods*. Data shown are the mean ± SD of at least three independent experiments, with ***p<0.001.

To determine the entry route used by ACT into the CR3**^−^** cells, we analysed the effects of several inhibitors of endocytosis on the ACT internalisation. Incubation of cells with dimethylamiloride (DMA), which blocks the entry by macropinocytosis [32] showed no blocking effect on the ACT uptake by the CHO-K1 cells (**Fig. 2A**). This entry pathway was thus dismissed. The effect induced by chlorpromazine, which induces the misassembly of clathrin-coated pits at the plasma membrane [33] was negligible as it was at the limit of the significance (**Fig. 2A**). Incubation of cells in a hyperosmotic medium (high-sucrose), which has also been probed to interfere with the clathrin-mediated endocytosis [34] neither showed any significant change (**Fig. 2A**). Transferrin uptake, which occurs constitutively via clathrin-mediated endocytosis [35], was used as control. The nearly complete elimination of the transferrin uptake after incubation of the CHO-K1 cells with concentrations of chlorpromazine (5 µg/ml) identical to those used in the assay with the toxin (**Fig. 2B**) made clear that the clathrin-coated pit endocytosis pathway was fully functional in these cells. Collectively these data demonstrate first, that ACT is endocytosed by non-phagocytic cells, and therefore, that interaction with its receptor, the CR3 integrin, is not required, and second, that the entry mechanism followed by ACT in the CHO-K1 cells is not clathrin-dependent.

An important inhibitory effect on the toxin uptake was observed upon preincubation of cells with nocodazole (microtubule-depolymerizing drug) and cytochalasin D (F-actin- depolymerizing drug) (**Fig. 2A**) indicating that an intact cytoskeleton, and likely, vesicle-mediated trafficking are involved in the ACT internalisation pathway.

### Caveolin-1-dependent Uptake of ACT in the CHO-K1 Cells

Another well-characterized endocytic pathway is caveolae-mediated [36], [37]. Caveolae are rich in cholesterol and sphingolipids [38], [39], and as lipid rafts, are sensitive to cholesterol-depleting drugs. Caveolin-1 is the major resident scaffolding protein constituent of caveolae [39], [40]. We addressed the possibility of a caveolae-mediated uptake for ACT in the CHO-K1 cells.

**Figure 3:**
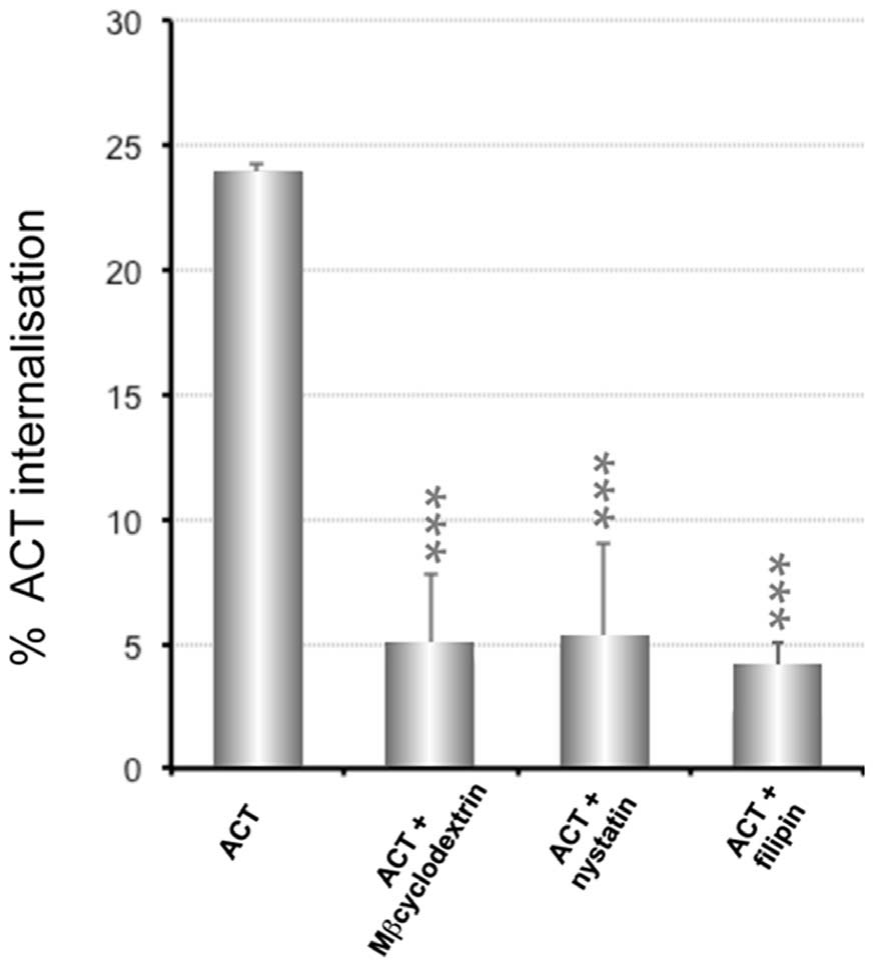
Effect of cholesterol depleting agents on the internalisation of ACT into CHO-K1 cells. CHO-K1 cells were pre-incubated for 30 min at 37°C with three different cholesterol depleting agents, methyl-β-cyclodextrin (10 mM), nystatin (0.25 µg/ml) and filipin (0.5 µg/ml). Cells were incubated with 35 nM ACT at 37°C for 10 min. Then, the surface staining of ACT was measured using a flow cytometer. Internalisation was assessed by FACS and expressed as described in *Materials and Methods*. Data shown are the mean ± SD of at least three independent experiments, with ***p<0.001.

We first evaluated the effect of three known lipid raft disrupting, cholesterol depleting agents, nystatin, filipin and methyl-β-cyclodextrin (MβCD) on the ACT internalisation into CHO-K1 cells. As shown in **Fig. 3** preincubation of cells with the three chemicals importantly inhibited ACT internalisation. This was thus a first indication that cholesterol-enriched membrane microdomains may be part of the scenario in the ACT endocytic process in the CHO-K1 cells. Specificity of nystatin, filipin and MβCD for non-clathrin pathways was tested, and as expected, it was observed that pre-incubation of the CHO-K1 cells with these three chemicals did not affect the endocytosis of transferrin (taken up by clathrin-coated vesicles), while the internalisation of the raft-marker BODIPY-lactosylceramide, which has been reported to be internalised via caveolae [39], substantially decreased in the pre-treated cells (**Figure S2**).

**Figure 4:**
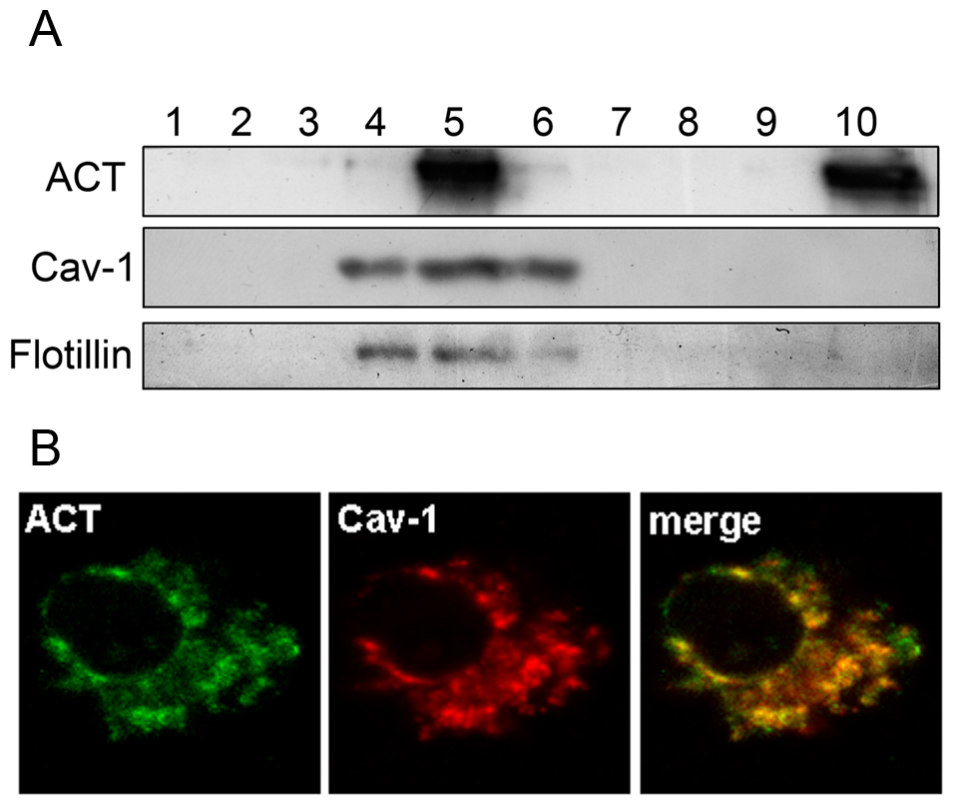
Western blot analysis of sucrose density gradient fractions and confocal microscopy images showing ACT localization in ACT-treated CHO-K1 cells. (A) Western blot analysis of sucrose density gradient fractions obtained by centrifugation of Triton X-100-solubilized CHO-K1 cells treated with 35 nM ACT showed that the toxin was recovered from high-buoyant density fractions of the gradient, and that it co-localized with caveolin-1 and flotillin. The procedure was performed as described in *Materials and Methods*. A representative experiment from three independently performed assays is shown. (B) Confocal microscopy analysis of the localization of ACT and Cav-1 in CHO-K1 cells. Cells were incubated for 10 min with the toxin, washed three times, fixed and permeabilized to analyze ACT and Cav-1 by immunohistochemistry. A representative image is shown.

Western blot analysis of sucrose density gradient fractions obtained from Triton X-100-solubilized CHO-K1 cells showed that part of ACT co-localized with caveolin-1 and flotillin in the ACT-treated cells (**Fig. 4A**). Resistance to solubilization by nonionic detergents at 4°C is a biochemical characteristic of rafts, which allows their purification on density gradients. Confocal microscopy images confirmed the co-localization of the toxin with caveolin-1 (**Fig. 4B**).

**Figure 5:**
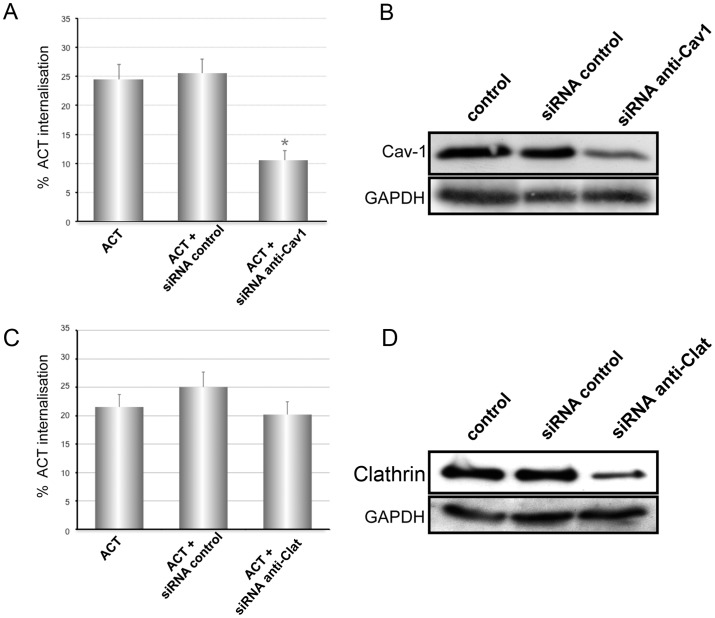
Knock down of caveolin-1 and and clathrin, and its effect on the ACT internalisation into CHO-K1 cells. Cells were transfected with anti-Cav-1 siRNA or with a non-effective scrambled vector (mock cells). Then, silenced or mock cells were incubated with 35 nM ACT at 37°C for 10 min and surface staining of ACT followed in a flow cytometer. Internalisation of ACT was determined by FACS as described in *Materials and Methods* (**A**). Western blotting detection of caveolin-1 protein expression in CHO-K1 cells after transfection with the siRNA molecules (**B**). Cells were transfected with anti-Clathrin siRNA or with a non-effective scrambled vector (mock cells). Then, silenced or mock cells were incubated with 35 nM ACT at 37°C for 10 min and surface staining of ACT followed in a flow cytometer. Internalisation of ACT was determined by FACS as described in *Materials and Methods* (**C**). Western blotting detection of clathrin expression in CHO-K1 cells after transfection with the siRNA molecules (**D**). The procedure was performed as described in *Materials and Methods*. Equal loading of protein was confirmed in each blot by membrane stripping and further incubation with antibodies to visualize cytosolic GAPDH protein (Lower panel of the figure). The extent of protein silencing was determined by quantitative densitometric analysis. Data shown in the left-hand panels of the figure are the mean ± SD of at least three independent experiments, with *p<0.001.

An independent experiment of caveolin-1 knock down further confirmed the hypothesis of a raft-dependent, caveolae-mediated endocytosis of ACT in the CHO-K1 cells. As shown in **Fig. 5A** the percentage of toxin internalisation was importantly reduced in CHO-K1 cells transfected with anti-Cav-1 siRNA. The specificity of the anti-Cav-1 siRNA action was confirmed by using a non-effective scrambled vector which did not show any effect on the ACT endocytosis (**Fig. 5A**). The control for the silencing of Cav-1 protein upon transfection of the anti-Cav-1 siRNA is shown in **Fig. 5B**. This figure also shows the null effect of the non-effective scrambled vector on the Cav-1 expression. In addition, clathrin expression was also silenced by transfection of the CKO-K1 cells with anti-clathrin siRNA. **Fig. 5C** shows that the silencing of clathrin did not have any effect on the ACT uptake by the CHO-K1 cells. A random siRNA was also tested to prove the specificity of the anti-clathrin siRNA used (**Fig. 5C**). A representative experiment to detect the level of protein expression upon transfection is shown in **Fig. 5D**.

Hence all the shown data indicate that ACT internalisation in CHO-K1 cells takes place through caveolae, which contrasts with that observed in the naturally CR3 bearing J774A.1 cells where ACT is majorly internalised via clathrin-coated pits, likely together with its integrin receptor [26].

### Involvement of Src Tyrosine Kinases and Cav-1 Phosphorylation in the ACT uptake by the CHO-K1 Cells

It is implied by several studies that phosphorylation of caveolin-1 at tyrosine 14 is an essential step for caveolae-mediated endocytosis [41]–[44]. The Src family of non-receptor tyrosine kinases has been involved in this modification [45]–[48]. We checked these events in the ACT-treated cells.

**Figure 6:**
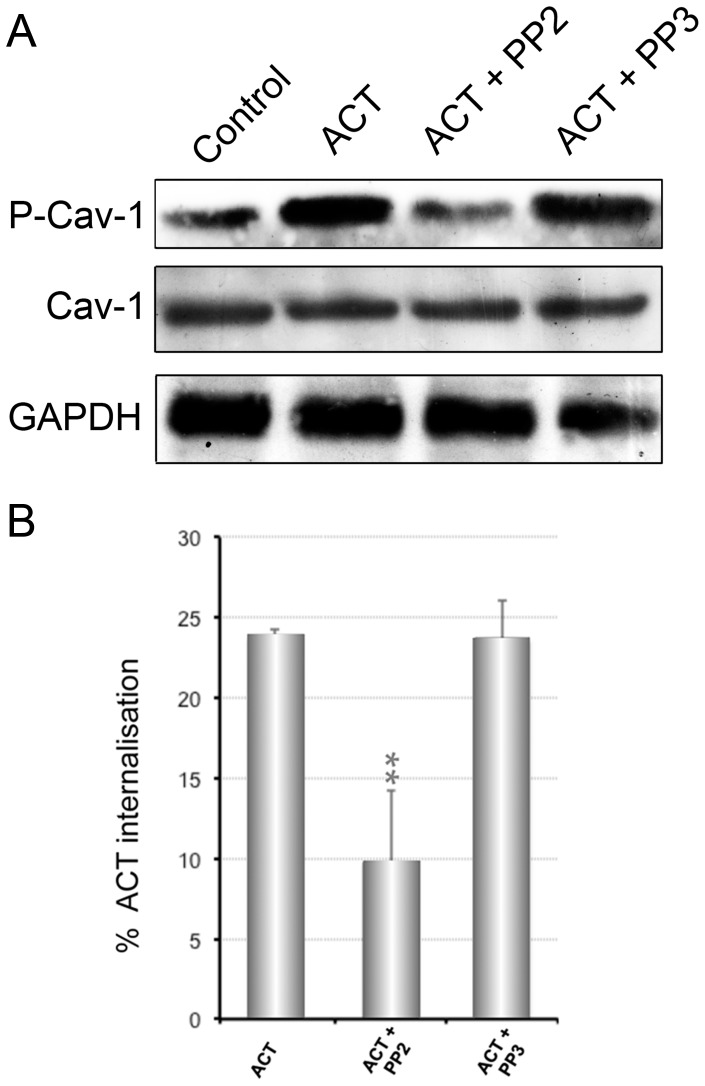
Phosphorylation of Cav-1 in ACT-treated CHO-K1 cells. Phosphorylation of Cav-1 in Tyr 14 was detected by Western blot of cell lysates incubated with the toxin (35 nM) for 5 min at 37°C (**A**). Caveolin-1 phosphorylation is significantly raised in the ACT-treated cells compared to control cells. ACT treatment raised about three times the Cav-1 phosphorylation in target cells. In addition, incubation of cells with PP2 (5 nM) significantly decreased the degree of Cav-1 phosphorylation, being similar to control cells. Incubation with PP3, the negative control of PP2, did not affect the Cav-1 phosphorylation induced by ACT treatment. Equal loading of protein was confirmed using specific antibodies to cytosolic GAPDH protein, after membrane stripping. Intensity of the bands was determined by scanning densitometry. (**B**) Effect of PP2 and PP3 on the ACT internalisation extent. CHO-K1 cells were pre-incubated for 30 min at 37°C with PP2 (5 nM) or PP3 (2,7 µM). Then cells were incubated with 35 nM ACT at 37°C for 10 min. Then, the surface staining of ACT was measured using a flow cytometer. Internalisation was assessed by FACS and expressed as described in *Materials and Methods*. Data shown in the lower panel are the mean ± SD of at least three independent experiments, with **p<0.025.

Phosphorylation of Cav-1 was followed by Western blot of cell lysates incubated with the toxin (35 nM) for 5 min at 37°C. An anti-P-Caveolin-1 antibody was used (abcam, Cambridge, UK). As shown in **Fig. 6A**, Caveolin-1 phosphorylation is very importantly raised in the ACT-treated cells as compared to control cells. The P-Cav-1/Cav-1 ratio in ACT treated cells was three-fold higher that in control cells (**Fig. 6A**). In addition, incubation of cells with PP2, a specific inhibitor of tyrosine kinases from the Src family, significantly decreased the degree of Cav-1 phosphorylation to control levels, while PP3, which is an inactive analogous of PP2, showed no effect (**Fig. 6A**). To determine whether the phosphorylation status of Cav-1 had indeed a direct effect on the ACT-internalisation in the CHO-K1 cells, we quantified the percentage of toxin internalisation in cells pre-incubated with PP3 and with PP2. As shown in **Fig. 6B**, PP2, the Tyr-kinase inhibitor, significantly decreased the ACT internalisation percentage, while PP3 had no effect. Together these results suggested that Cav-1 phosphorylation by Src tyrosine kinases might be likely involved in a caveolae-mediated ACT uptake by CHO-K1 cells.

### ACT and its Receptor, the Integrin CR3, are Internalised by CR3^+^CHO-K1 Transfected Cells

**Figure 7:**
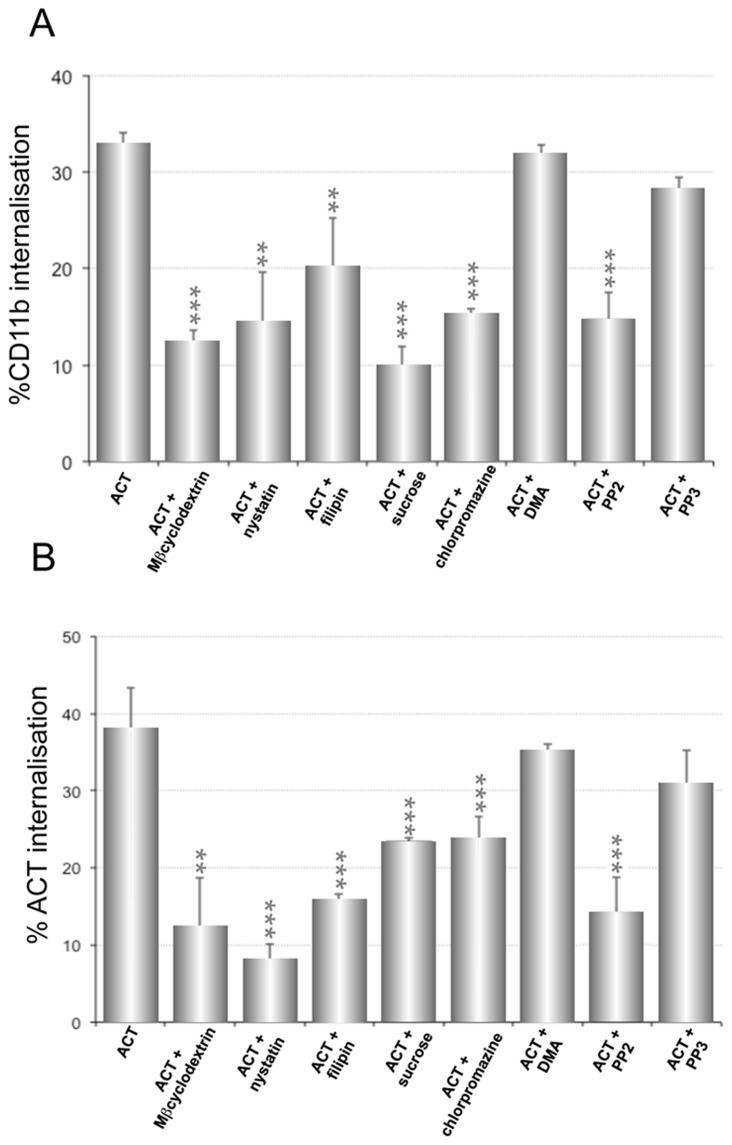
ACT and its CD11b/CD18 receptor are internalised by CR3^+^CHO-K1 cells. CHO-K1 cells stably transfected to express CD11b and CD18 (CR3^+^CHO-K1 cells) and mock control cells were used to assess internalisation of ACT and of its receptor, the integrin CD11b/CD18 or CR3, as well as to test the effect of several inhibitors into the ACT-induced internalisation processes. The concentrations of the different compounds, used in these experiments, are identical to those used in the former experiments. (**A**) Internalisation of the CD11b/CD18 integrin by the CR3^+^CHO-K1 cells. (**B**) ACT internalisation. Internalisation was determined by FACS as described in *Materials and Methods*. Data shown are the mean ± SD of at least three independent experiments, with **p<0.025 and ***p<0.001.

**Figure 8:**
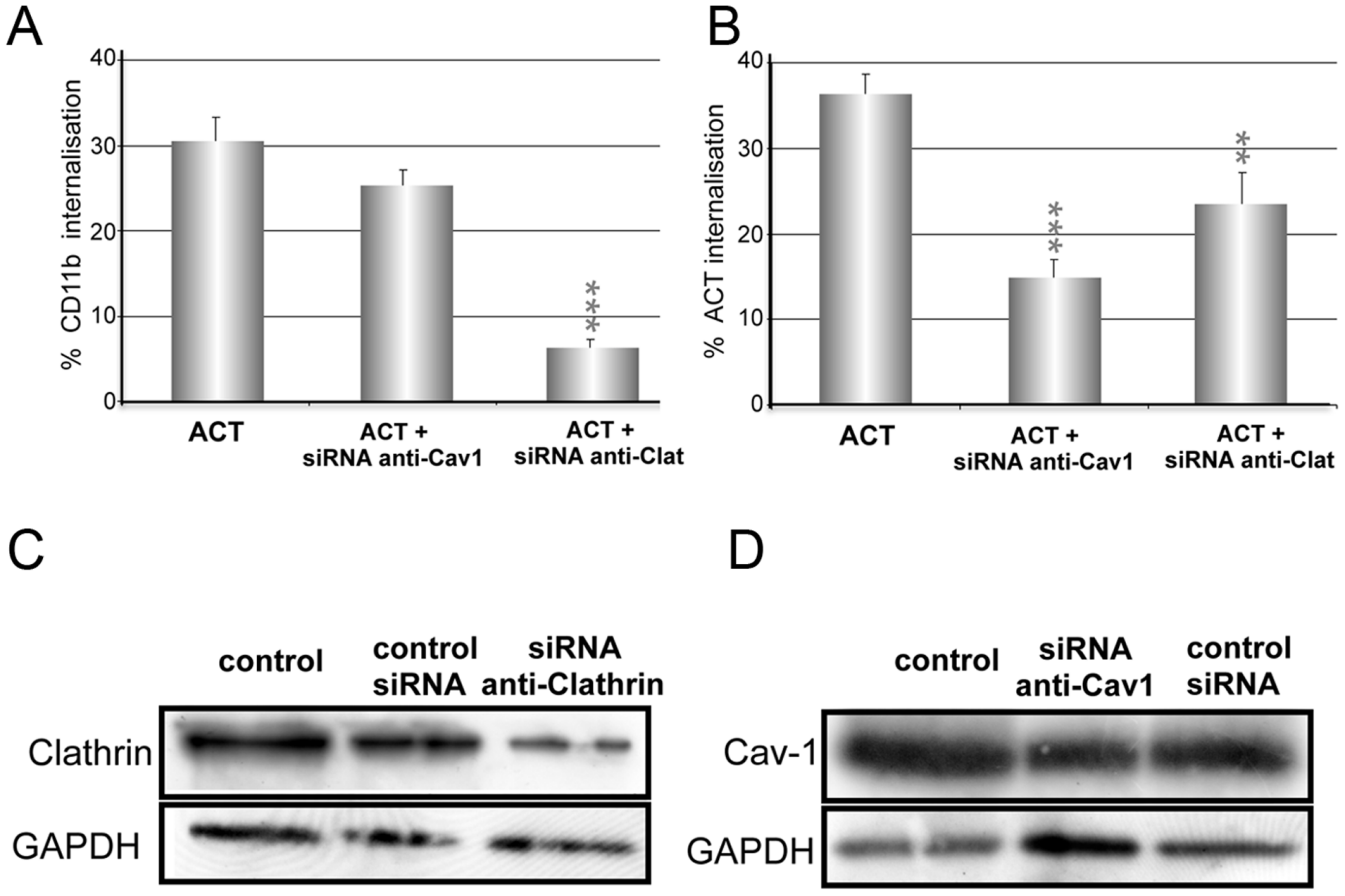
Knock down of caveolin-1 or clathrin, and its effect on the ACT internalisation into the CR3^+^CHO-K1 cells. A knockdown experiment, in which expression of Cav-1 and clathrin was silenced by the corresponding siRNA molecules, was performed in the CR3^+^CHO-K1 cells. Then, the silenced or control mock cells were incubated with ACT at 37°C and surface staining of ACT followed in a flow cytometer. (**A**) Extent of CD11b internalisation upon silencing the expression of Cav-1 or clathrin, (**B**) Extent of ACT internalisation upon silencing the expression of Cav-1 or clathrin, Internalisation was determined by FACS as described in *Materials and Methods*. (**C**) Western blotting detection of clathrin protein expression in CR3^+^CHO-K1 cells after transfection with anti-siRNA. (**D**) Western blotting detection of caveolin-1 protein expression in CR3^+^CHO-K1 cells after transfection with anti-cav-1 siRNA molecules. Extent of protein silencing was determined as described in the legend of Fig. 5. Data shown in the upper panels are the mean ± SD of at least three independent experiments, with **p<0.025, ***p<0.001.

We previously reported that in the naturally CR3 expressing J774A.1 macrophage cell line ACT induces a rapid internalisation of the integrin [26]. Here we used CHO-K1 cells stably transfected to express CD11b and CD18 (CR3^+^CHO-K1 cells) to check whether ACT was able to trigger internalisation events. The level of the CR3 integrin expression in these transfected cells was measured by flow cytometry and it was found to be similar to the amount of integrin expressed in the J774A.1 cell line (**Figure S3**). As shown in **Fig. 7A** incubation of CR3^+^CHO-K1 cells with ACT (35 nM) at 37°C induced about 35% internalisation of the β2 integrin, a value very close to the reported for J774A.1 macrophages [24]. Chlorpromazine and high sucrose medium very importantly diminished the uptake of CR3 in these cells (≈ 60–70%) (**Fig. 7A**). Cholesterol-depleting agents also induced a decrease of the integrin uptake, but the diminishment was less pronounced than the observed for ACT in the non-transfected CHO-K1 cells (see **Fig. 3**), suggesting that the β2 integrin majorly follows a clathrin-dependent endocytosis in the CR3^+^ transfected cells. Consistent with this, a nearly complete elimination of the ACT-induced β2 integrin endocytosis was observed in CR3^+^CHO-K1 cells in which clathrin expression was silenced by an anti-clathrin siRNA, whereas the effect of silencing the expression of Cav-1 on the integrin uptake was not significant (**Fig. 8A**). Controls of silencing of clathrin and Cav-1 expression by the corresponding siRNA are shown (**Fig. 8C and 8D**).

The toxin itself was also internalised into the CR3^+^CHO-K1 cells (**Fig. 7B**). The percentage of ACT endocytosed after 10 min at 37°C was of about 35–40% of the total toxin bound to the cells (**Fig. 7B**). In this case, chlorpromazine pre-incubation and high sucrose medium induced a more moderate decrease of the percentage of ACT internalised (about 40%) (**Fig. 7B**) as compared to the effect induced by the same chemicals on the integrin endocytosis (≈ 60–70%) (**Fig. 7A**). The cholesterol-depleting chemicals (nystatin, filipin and MβCD) were however more effective than in the case of the integrin hampering the toxin uptake. These data suggested that the toxin might follow more than one entry pathway in the transfected CHO-K1 cells. This hypothesis was confirmed by a knockdown experiment, in which expression of Cav-1 or clathrin was silenced by the corresponding siRNA. As shown in the figure **Fig. 8B** either the silencing of clathrin as the silencing of Cav-1 significantly reduced the toxin uptake. The corresponding controls of protein expression in the knockdown assays are shown (**Figs. 8C and 8D**).

Finally we checked the participation of tyrosine kinases in the entry process. As shown in **Fig. 7A and 7B**, either the internalisation of the CR3 integrin, as of the toxin, showed to be sensible to PP2 (specific inhibitor of the Src tyrosine kinases) and insensible to PP3 (inactive analogous), suggesting therefore involvement of non-receptor tyrosine kinases in the endocytosis of ACT and of the CR3 integrin in the CR3^+^CHO-K1 cells.

### ACT Activates a Clathrin-dependent, but Receptor-independent, Endocytic Pathway in CR3^−^J774A.1 Cells

We used J774A.1 cells in which expression of the CD11b chain of the integrin was silenced by an anti-CD11b shRNA (CR3**^−^**J774A.1 cells) to unravel the influence of cell physiology and characteristics in the uptake of ACT **(**quantification of the CR3 knock down extent is shown in **(Figure S1)**.

**Figure 9:**
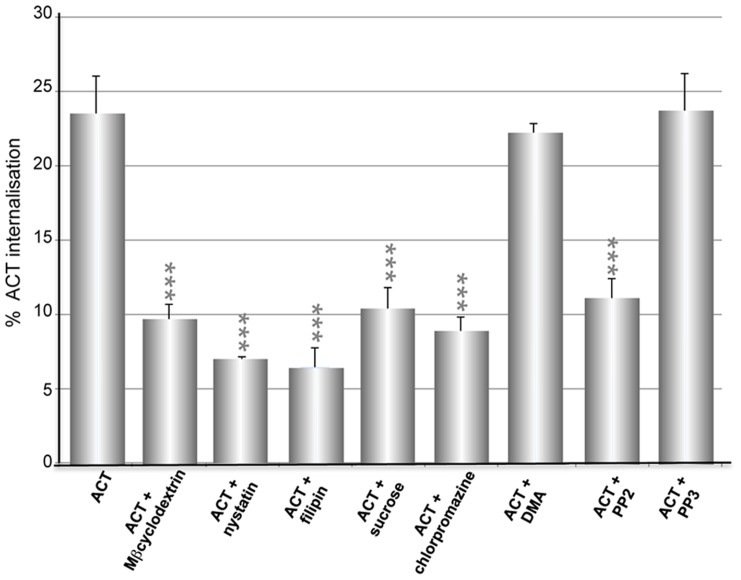
ACT is endocytosed by clathrin-dependent, but receptor-independent, pathway in CR3^−^J774A.1 cells. J774A.1 cells in which CD11b expression was silenced by an anti-CD11b shRNA were used to assess ACT internalisation into these CR3**^−^** macrophages, as well as to test the effect of several inhibitors into this internalisation. The concentrations of the different compounds, used in these experiments, are identical to those used in the former experiments. Internalisation was determined by FACS as described in *Materials and Methods*. Data shown are the mean ± SD of at least three independent experiments, with ***p<0.001.

Firstly we determined that ACT was also endocytosed by these CR3**^−^** cells **(** **Fig. 9** **)**, with a maximum extent of internalisation of about 25% after 10 min of incubation with the toxin, which is below to the observed previously in the J774A.1 cells (≈35–40%) [26]. A time-course of the toxin internalisation in this cell line is shown in the **Figure S4**. A lower toxin entry percentage is nevertheless consistent with an expected lower binding of the toxin to these CR3-negative cells, which do not express the toxin receptor [26]. The toxin uptake was importantly abolished by inhibitors of clathrin-dependent endocytosis, such as chlorpromazine and high sucrose medium (**Fig. 9**). This suggests that even though there is nearly no receptor present on the cell surface, the clathrin-dependent endocytosis pathway can be activated in this CR3**^−^** cell line by ACT action, suggesting therefore that the cell physiology and characteristics may indeed dictate the route of entry for the ACT. Nevertheless we cannot rule out totally that another cholesterol-sensitive entry mechanism also intervene in the uptake of part of the toxin, as ACT uptake was affected by cholesterol-depleting drugs (**Fig. 9**), while transferring endocytosis, which takes place only through clathrin-coated vesicles was not (**Figure S5**).

**Figure 10:**
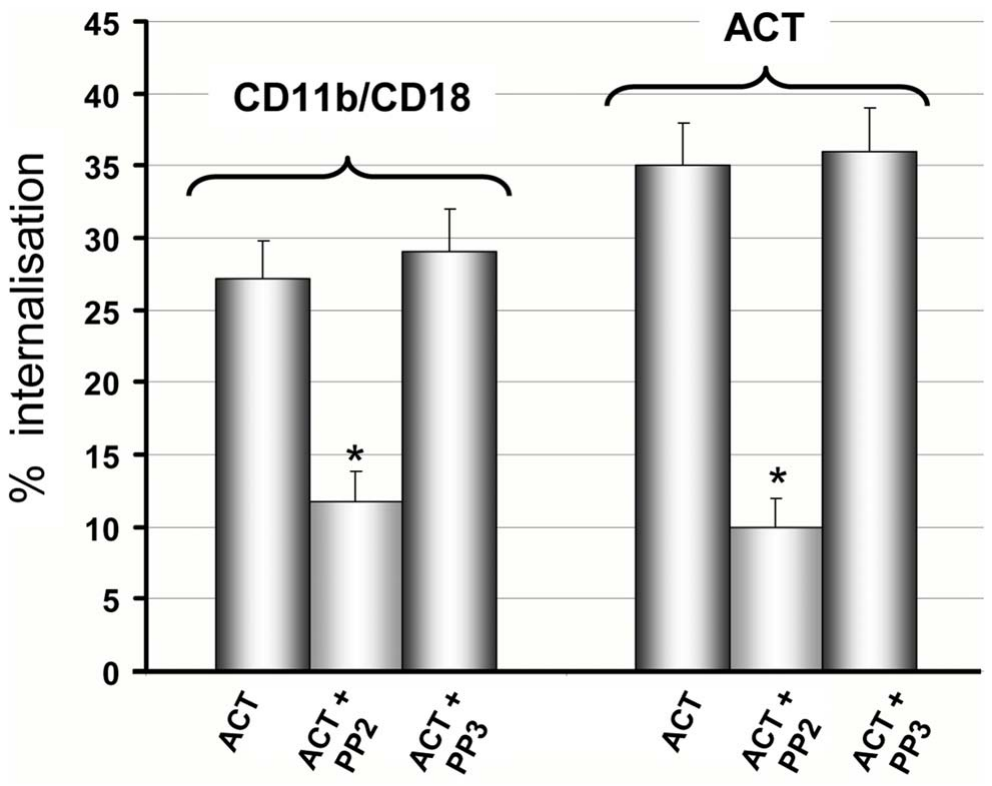
Endocytosis of ACT and its CD11b/CD18 receptor is mediated by Src Tyr kinases in J774A.1 cells. J774A.1 cells were pre-incubated for 30 min at 37°C with PP2 to inhibit Src Tyr Kinases and PP3 as negative control for PP2. Then ACT was added at a final concentration of 35 nM, and cells further incubated at 37°C for 10 min. Internalisation of ACT and CD11b was assessed by FACS and expressed as described in *Materials and Methods*. Data shown are the mean ± SD of at least three independent experiments, with *p<0.001.

As observed in the CHO-K1 and transfected CR3^+^CHO-K1 cells, PP2 (specific inhibitor of the Src tyrosine kinases) was effective inhibiting the ACT uptake, while to PP3 (inactive analogous was not (**Fig. 9**) suggesting once again the participation of non-receptor Tyrosine kinases in the endocytosis of ACT in the CR3**^−^**J774A.1 cells. As comparison, the effect of PP2 and PP3 on the toxin uptake by the naturally CD11b expressing J774A.1 macrophages is shown in **Fig. 10**. Hence, it is proved that the activation of non-receptor Src Tyr kinases, by the toxin action, represents a common mechanism in the endocytic pathways activated by this toxin, either in CD11b-positive as in- negative cells.

### ACT-induced Ca^2+^ Influx Triggers the Toxin Uptake by the Different Target Cells

**Figure 11:**
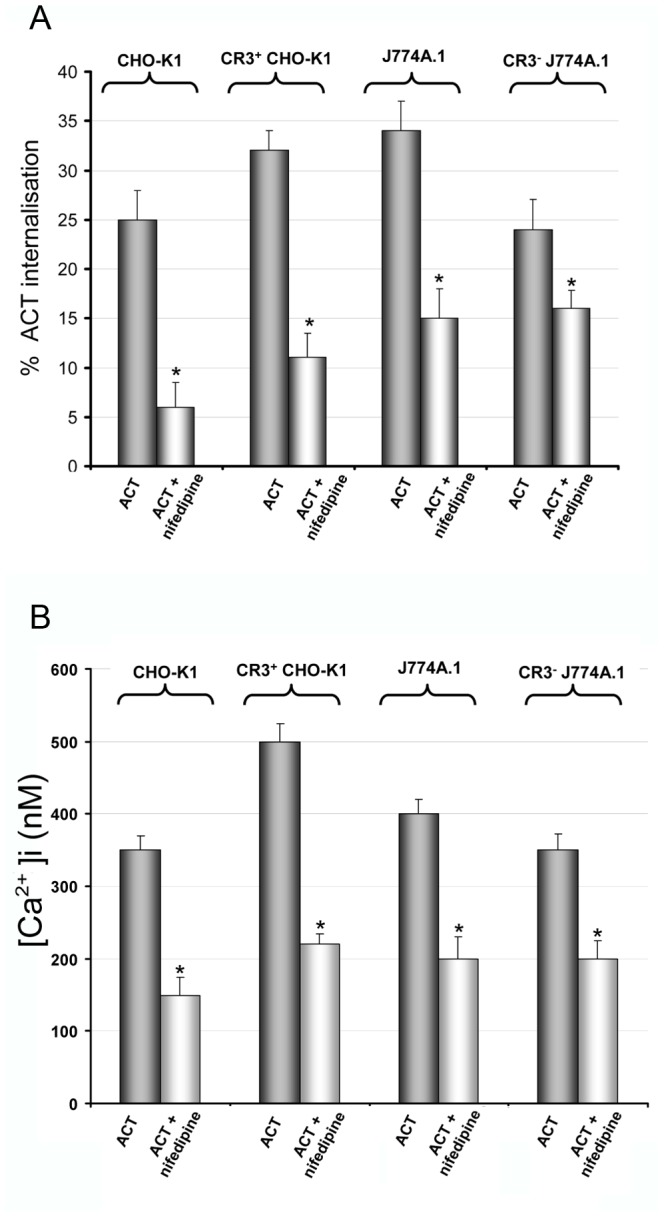
ACT-induced Ca^2+^ influx triggers the toxin uptake by the different target cells. (**A**) Pre-incubation of the different cells with nifedipine (10 µM), which was previously shown to effectively block the ACT-induced Ca^2+^ influx [18], very importantly decreased the toxin uptake by either CHO-K1 cells, as CR3^+^CHO-K1 cells or by J774A.1 and CR3**^−^**J774A.1 cells, hence suggesting that Ca^2+^ influx induced by ACT is a common master denominator in the different endocytosis routes used by the different cells to take the toxin. Cells were preincubated 30 min at 37°C, then 35 nM ACT was added and cells further incubated for 10 min. Internalisation was determined by FACS as described in *Materials and Methods*. Data shown are the mean ± SD of at least three independent experiments, with *p<0.001. (**B**) The intracellular calcium level was measured using FURA-2, as described in the *Methods* section, in the different cell lines, in presence or absence of nifedipine. Data shown are the mean ± SD of at least three independent experiments, with *p<0.001.

Previously we had provide convincing biochemical, biophysical and cell biology data to show that ACT and integrin molecules, along with other raft components, are rapidly internalised by the macrophages in a toxin-induced calcium rise-dependent process [26]. Here, we explored whether the toxin-induced calcium elevation is also involved in triggering the toxin uptake by the other cell types used in the present study. We first corroborated that the toxin induces similar calcium elevations in every cell lines used here. **Fig. 11** and **Figure S6** show the results corresponding to the CR3^+^CHO-K1 and CR3**^−^**J774A.1 cells. The data corresponding to J774A.1 macrophages and CHO-K1 cells were already reported in a previous work from our laboratory [18].

To test the link between the ACT-induced calcium entry and ACT-triggered endocytosis, the different cells were pre-incubated with nifedipine, which was previously shown to cause inhibition of the ACT-induced Ca^2+^ influx [18]. It was observed that this inhibitor of L-type calcium channels, decreased significantly the toxin uptake by either CHO-K1, as CR3^+^CHO-K1 or by J774A.1 and CR3**^−^**J774A.1 cells (**Fig. 11A** **)**, hence suggesting that Ca^2+^ influx induced by ACT is as well another common denominator in the different endocytosis routes used by the different cells to take the toxin.

### Toxin Endocytosis Involved in the Repair of Plasma Membrane Lesions Caused by ACT

**Figure 12:**
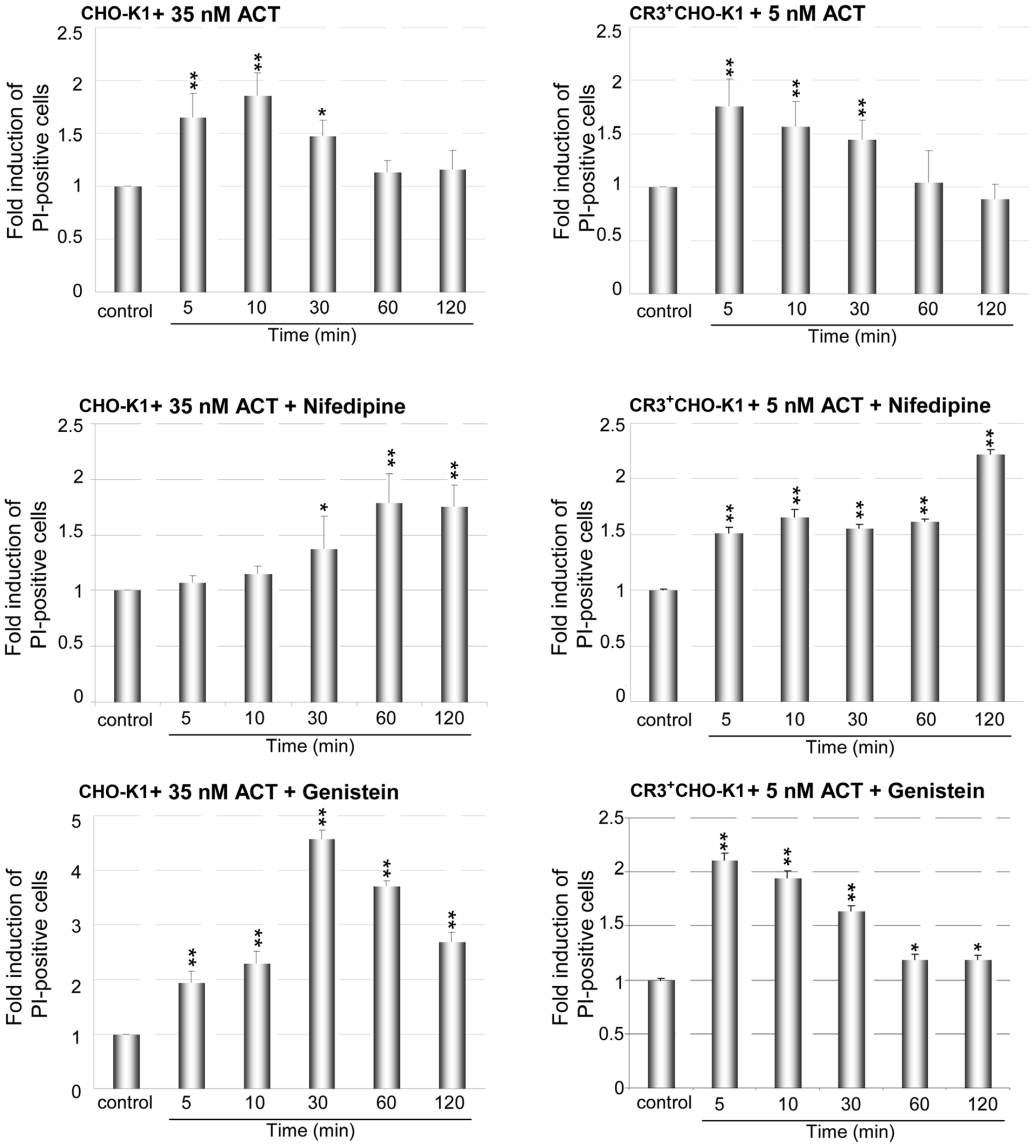
Uptake of propidium iodide (PI) by ACT-treated CHO and CR3^+^CHO-K1 cells. CHO cells were treated with 35-CR3 cells with 5 nM ACT for different incubation times (5, 10, 30, 60 and 120 min), then washed, trypsinized and incubated with propidium iodide for 4 min. PI uptake by the different cells was determined by flow cytometry as described in Materials and Methods section. Population of viable cells which show PI staining were gated in a FSH *vs* PI density plot, and propidium iodide positive cells were enumerated; data are displayed as fold activation over untreated controls with data-points representing mean values of at least three independent experiments ± SEM of at least three independent experiments, with **p<0.025, ***p<0.001.

Several previous studies had documented the capacity of nucleated mammalian cells to cope with a limited number of membrane lesions caused by pore-forming toxins, and recover, in some cases through a membrane-repair response which involves the endocytosis of the toxin from the membrane. The mechanisms involved in the cell recovery process are diverse yet specific [27]–[30]. Hence, we tested here whether the different cells used were capable of repairing the lesions created by ACT at the plasma membrane. Recovery of the plasma membrane integrity was followed by FACS quantification of propidium iodide (PI) uptake by the cells, upon incubation with the toxin. **Fig. 12** shows that after a short time upon toxin incubation (5–10 min) with either CHO-K1 cells as with CR3^+^CHO-K1 cells, PI could penetrate into the treated cells, suggesting membrane damage by permeabilization, likely through pore formation. However, after longer periods (1 hour) PI was excluded, hence revealing a rapid recovery of the membrane integrity (**Fig. 12**). The good temporal correlation between the ACT uptake by the two cell types assayed and the kinetics of plasma membrane recovery shown in **Fig. 12** supports the idea that elimination of toxin molecules from the cell membrane by endocytosis may be indeed involved in the repair response to cope with the toxin insult.

As shown in the previous sections, two key factors in ACT internalisation are the tyrosine kinases and the intracellular calcium elevation. To unravel whether these two factors may be directly linked to the repair response observed here, cells were pre-treated with genistein, inhibitor of Tyr kinases, or with nifedipine, inhibitor of L-type Ca^2+^ channels, and then, the PI uptake determined.

The effect of genistein in the CHO-K1 cells was to increase (up to five times) the uptake of PI by the ACT-treated cells, at short times (**Fig. 12**), indicating a higher membrane permeabilization. The more (pore-forming) toxin molecules remain in the membrane, the higher is the permeability. It can be inferred thus that toxin elimination by endocytosis (clathrin/caveolae-dependent) is indeed involved in membrane repair. Genistein does not block totally the toxin uptake, and in consequence the cells still show signs of recovery at longer times.

In the cells pre-treated with nifedipine the response was more complex. Firstly, cells took longer to become permeable to the propidium iodide (almost 60 min in the CHO-K1 cells), and after that time, the cells did not apparently recover, remaining permeable to PI (**Fig. 12**). In the light of these results, and basing on recent data from our laboratory [49], we hypothesize that calcium influx is necessary for two different steps in the toxin context. We have demonstrated that ACT is processed at its N-terminus by calcium-activated cellular calpains. As result of the cleavage the N-terminal adenylate cyclase domain is released into the cytosol, while the C-terminal (pore-forming) RTX domain remains in the membrane. An ACT-induced calcium rise is absolutely required for the toxin processing [49], and hence, it is also required for the accumulation of the “pore-forming” RTX fragments in the plasma membrane. If the accumulation of the “pore-forming” fragments of ACT is hampered, as by incubating the cells with nifedipine, then less permeabilization would be expected and concomitantly, less PI uptake. This could explain the delay in the PI uptake observed in the ACT-treated CHO-K1 cells pre-treated with nifedipine (**Fig. 12**). On other side, the lower permeabilization observed in these nifedipine-pretreated cells could respond to a lower lytic capacity of the unprocessed ACT as compared to the processed one. Finally, the inhibitory effect of nifedipine on ACT internalisation **(** **Fig. 11** **)** also suggests that a calcium-triggered repair-process may be underlying the cell survival.

## Discussion

We present here a detailed study addressing the endocytosis of ACT in four different cell types, CHO-K1 cells, CR3^+^CHO-K1 cells, in which the CR3 has been stably transfected, J774A.1 cells naturally expressing CR3 and CR3**^−^**J774A.1 cells, macrophages in which expression of the CD11b chain has been knocked down. And we show that ACT is rapidly eliminated from the cell membrane either in CR3-positive as negative cells, though through different routes of entry depending on the cell type. Two key factors, the activation of non-receptor Tyrosine kinases, and the elevation of the intracellular calcium, both induced by ACT action, are involved in the ACT endocytosis pathways.

In the non-immune cells devoid of the toxin receptor (CHO-K1 cells) ACT is taken up by caveolae-mediated endocytosis. Evidences of that are the null effect of chlorpromazine and high sucrose medium, the substantially reduced toxin entry in cholesterol-depleted conditions, the co-localization of the ACT with the protein caveolin-1 and the reduced toxin entry in the Cav-1 knock-down cells. Interestingly, the expression of the toxin receptor (integrin CR3) on the surface of the transfected CR3^+^CHO-K1 cells has several consequences in the toxin uptake by these non-immune cells. One is that, besides activating the caveolae-dependent route, the clathrin-dependent pathway is also activated. A second consequence is that the integrin itself is also endocytosed, majorly by clathrin-coated vesicles, and a third one is that in presence of the receptor the toxin enters the target cells through the two known routes of entry, caveolae-dependent and clathrin-dependent. This suggests that the presence of the integrin, somehow imposes to part of the toxin (roughly half) to follow the clathrin-dependent entry pathway. It can be conceived that the toxin molecules that enter cells through this pathway likely correspond either to toxin molecules that still remain associated to receptor molecules, or to toxin molecules dissociated from the receptor, but still in close proximity to it. Thus, the binding or proximity to the receptor would make the toxin to be internalised along with it through clathrin-coated vesicles. On the contrary, the ACT molecules that are taken up though caveolae might likely correspond to toxin molecules very likely localized in raft-like membrane domains, perhaps by binding to these cells independently of the receptor.

In contrast, in the knock-down CR3**^−^**J774A.1 cells, which do not virtually express the integrin on its surface, but conserve the macrophage phenotype, the toxin is majorly taken up through clathrin-coated vesicles. This suggests that binding to the receptor is not required necessarily to activate a clathrin-dependent ACT internalisation in the macrophages, and hence, that the cell specific physiology and characteristics may be also an important determining factor in the activation of a particular endocytic route in the ACT-treated cells.

We show here that the elevation of intracellular Ca^2+^ concentration induced by ACT is fundamental in the toxin uptake by all the cell types studied here. An inter-relation between calcium elevations and endocytosis processes has been widely documented. That Ca^2+^ influx through Ca^2+^ -channels can trigger endocytosis is a well established mechanism in non-excitable cells, such as oocytes [50]. A biological response involving Ca^2+^-activated endocytosis has also been reported to be the response of cells to membrane ruptures induced either by mechanical forces as well as by a few pore-forming proteins. The large pore-forming toxin SLO, the small pore-forming toxin α-toxin, and medium sized perforin trigger endocytic events which correlate with plasma membrane resealing [26], [27], [51]. It has been shown that, Ca^2+^ influx through the “holes” on the cell membrane triggers resealing of the membrane wounds by fusion of internal membranes with the cell surface, followed by removal of “lesion” membrane by endocytosis [28]. Here, we provide evidences that ACT-damaged cells are recovered from the toxin insult. We show that shortly after incubation with the toxin (≈5 min) ACT-treated cells become permeable to propidium iodide, suggesting a rapid membrane permeabilization, very likely through pore-formation. After a longer period of time (≈1 hour) the cells become progressively impermeable to propidium iodide, hence indicating a recovery of the membrane integrity. A good temporal correlation is observed here among the “membrane repair process”, the toxin internalisation and the toxin-induced calcium elevation [18] and additionally it is observed that nifedipine pre-treatment of the cells, which inhibits the calcium entry and the ACT endocytosis, also interferes with the membrane recovery. Hence, these data support the notion that a calcium-mediated response involving the elimination of the toxin by endocytic processes might be directly link with the restoration of the plasma membrane integrity, observed here in the ACT-treated cells.

For some of the calcium-activated endocytosis events correlated with membrane resealing processes it has been shown the involvement of clathrin-coated vesicles, such as in the case of perforin lesions [51]. Here we show that ACT can be internalised by clathrin-dependent and caveolae-dependent pathways, which might also be part of the calcium-mediated endocytosis of ACT.

We also show that the activation of non-receptor Src Tyr kinases by ACT action is other instrumental factor involved either in the caveolae-dependent as in the clathrin-dependent endocytosis of the ACT, and linked also to the membrane recovery, as suggest our data in presence of inhibitors of tyrosine kinases (genistein). In fact, we observe that propidium iodide uptake is enhanced in presence of this inhibitor (genistein), suggesting that the more molecules remain in the membrane (by inhibition of the internalisation), the higher is the permeability to PI.

We hypothesize that the role played here by the ACT-triggered signalling involving tyrosine kinases and protein kinases such as the cAMP-dependent PKA may be to participate in the phosphorylation of different cellular substrates involved in the endocytic pathways, and that the particular cellular uptake pathway that will be activated by this ACT-triggered signalling may depend on the cell type physiology and characteristics and, on the specific cell substrates that may result phosphorylated by the kinases. Deciphering the identity of all the different protein targets that may result phosphorylated by ACT signalling is beyond the scope of the present study, yet, the finding of caveolin-1 phosphorylation in the ACT-treated CHO-K1 cells supports this idea.

Strikingly, in presence of nifedipine, cells require firstly, longer times to become visibly permeable to the propidium iodide (almost 60 min in the CHO-K1 cells), and after this time do not apparently recover, remaining permeable to PI. Relying on recent data from our laboratory, we hypothesize that calcium influx might be involved in two different, subsequent, steps in the toxin context. ACT-induced calcium elevation activates cellular calpains, which in turn cleave off the toxin’s N-terminal domain, releasing into the cytosol a soluble adenylate cyclase domain, while the C-terminal lytic (pore-forming) RTX domain remains in the membrane [49]. Nifedipine-sensitive ACT-induced calcium rise is absolutely required for this toxin processing. Accordingly, we postulate that the delay on the membrane permeabilization observed in the presence of nifedipine would be due to the hampering of the toxin cleavage and consequently, the hampering of the accumulation of the lytic “pore-forming” toxin fragments on the cell surface. Accordingly, the first step leading to the membrane permeabilization could be the toxin processing and subsequent accumulation in the membrane of “effective lytic ACT-pores”. This step could be followed by the activation of a calcium-mediated endocytic process aimed to reseal the membrane.

Several pore-forming toxins from the RTX family induce calcium elevations into the target cells (α-haemolysin from *E. coli*, leukotoxins from *Pasteurella haemolytica* and *Actinobacillus actinomytemcomitans*). It is therefore very tempting to bet that the rest of the pore-forming RTX toxins might also be capable of triggering survival membrane repair responses similar to the one shown here for *Bordetella* adenylate cyclase. In conclusion, our study provides a new example that supports and reinforces the recent idea that cells do not just swell and lyse, but are able to react to pore formation, mount a defense, even repair the damaged membrane and thus survive.

## Supporting Information

Figure S1 **Control of the CD11b silencing efficiency in CR3^−^J774A.1 transfected cells.** Surface expression of the CR3 integrin was detected by flow cytometry in CR3**^−^**J774A.1 cells and in the naturally CR3 expressing J774A.1 macrophages. Geometric mean fluorescence intensity (GMFI) was used to show CR3 expression. Data shown are the mean ± SD of at least three independent experiments, with *p<0.001.
(TIF)

Figure S2 **Effect of cholesterol-depletion on the internalisation of transferrin and BODIPY-LacCer by CHO-K1 cells.** CHO-K1 cells were pre-incubated for 30 min at 37°C with three different cholesterol depleting agents, methyl-β-cyclodextrin (10 mM), nystatin (25 µg/ml) and filipin (3 µg/ml). (A) Internalisation of FITC-transferrin with or without cholesterol depleting agents. (B) Internalisation of BODIPY-lactosylceramide with or without cholesterol depleting agents. Internalisation was assessed by FACS as described in *Materials and Methods*. Data shown are the mean ± SD of at least three independent experiments, with *p<0.001 respect to non treated cells.
(TIF)

Figure S3 **Control of effectiveness of CR3 transfection in CHO-K1 cells.** Surface expression of the CR3 integrin was detected by flow cytometry in control CHO-K1 cells, in the transfected CR3^+^CHO-K1 cells and in the naturally CR3 expressing J774A.1 macrophages. Geometric mean fluorescence intensity (GMFI) was used to show CR3 expression. Data shown are the mean ± SD of at least three independent experiments.
(TIF)

Figure S4 **Time-course of ACT internalisation in CR3^−^J774A.1 cells.** Addition of ACT (35 nM) to CR3**^−^**J774A.1 cells results in time-dependent internalisation of the toxin, as determined by flow cytometry and described in *Materials and Methods*. Geometric mean fluorescence intensities (GMFI) were used to show ACT internalisation. The data shown are the mean ± SD of at least three independent experiments, with ***p<0.001.
(TIF)

Figure S5 **Effect of cholesterol-depletion on the internalisation of FITC-Transferrin by CR3^−^J774A.1 cells.** CR3**^−^**J774A.1 cells were pre-incubated for 30 min at 37°C with three different cholesterol depleting agents, methyl-β-cyclodextrin (10 mM), nystatin (25 µg/ml) and filipin (3 µg/ml). Internalisation of FITC-transferrin with or without cholesterol depleting agents was assessed by FACS as described in *Materials and Methods*. Data shown are the mean ± SD of at least three independent experiments.
(TIF)

Figure S6 **Kinetics of calcium influx as determined by FURA-2 fluorescence in CR3^−^J774A.1 and CR3^+^CHO-K1 cells.** Calcium influx into (A) CR3^+^CHO-K1 and (B) CR3**^−^**J774A.1 cells was measured as previously described [18]. Briefly, cells grown on glass coverslips, were loaded with 2 µM fura2-AM for 30–45 min. The coverslips were mounted on a termostatized perfusion chamber on a Nikon Eclipse TE 300 based micro-spectrofluorometer and visualized with a ×40 oil-immersion fluorescence objective lens. At the indicated time, 35 nM ACT was added and the intracellular Ca^2+^ levels were determined using the method of Grynkiewicz et al. [31]. The ratio of light exited at 340 nm to that at 380 was determined with a Delta-Ram system (Photon Technologies International, Princeton). When required, cells were pre-incubated for 30 min with nifedipine, and then ACT was added. Data shown are the mean ± SD of three independent experiments.
(TIF)
