# Supplementary figures and images for: Ca2+ Influx and Tyrosine Kinases Trigger Bordetella Adenylate Cyclase Toxin (ACT) Endocytosis. Cell Physiology and Expression of the CD11b/CD18 Integrin Major Determinants of the Entry Route
Source: PLoS One. 2013 Sep 13;8(9):e74248. doi: 10.1371/journal.pone.0074248 (PMC3772820; doi:10.1371/journal.pone.0074248)

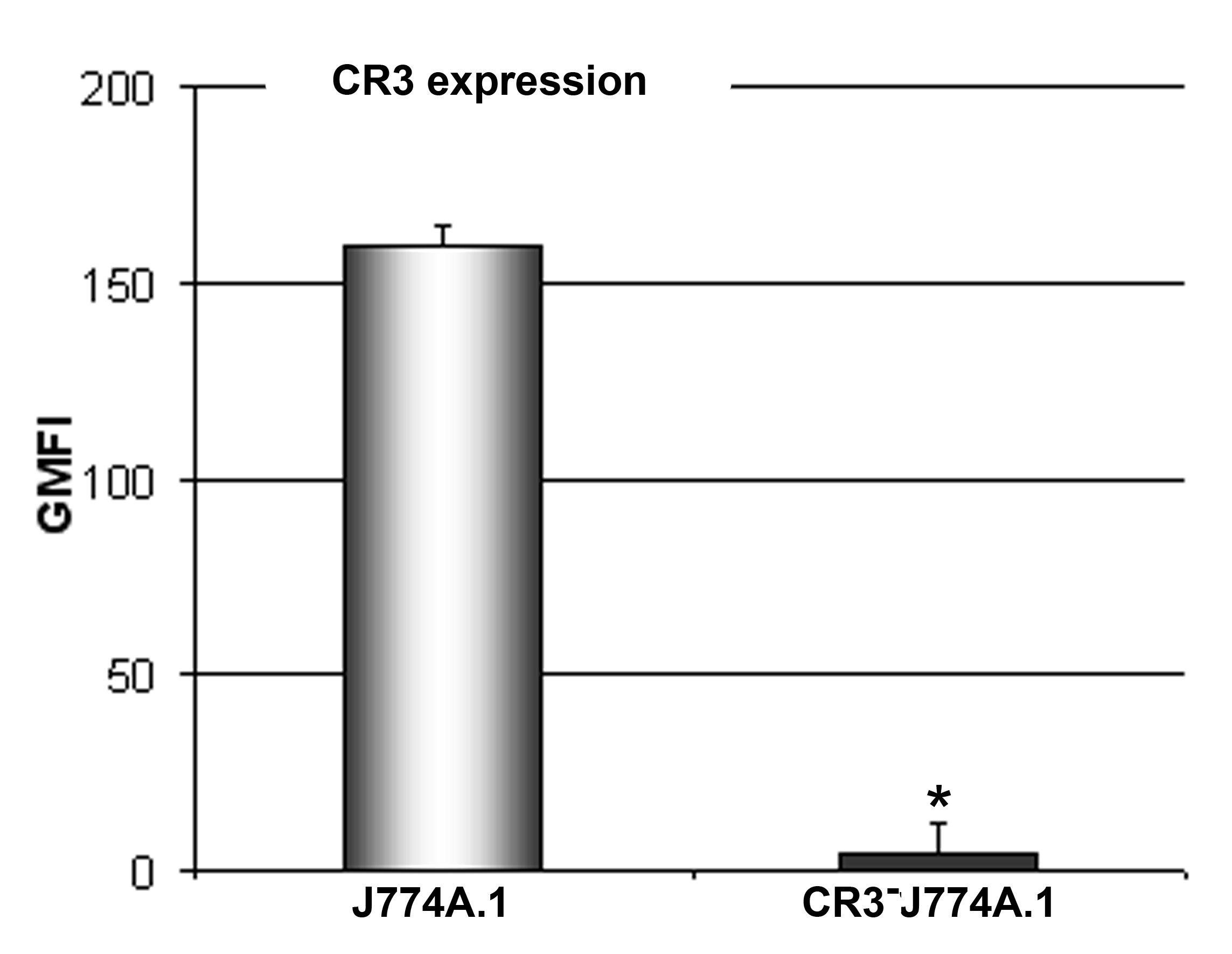

Supplement: Figure S1 — Control of the CD11b silencing efficiency in CR3−J774A.1 transfected cells. Surface expression of the CR3 integrin was detected by flow cytometry in CR3−J774A.1 cells and in the naturally CR3 expressing J774A.1 macrophages. Geometric mean fluorescence intensity (GMFI) was used to show CR3 expression. Data shown are the mean ± SD of at least three independent experiments, with *p<0.001. (TIF) [file pone.0074248.s001.tif]

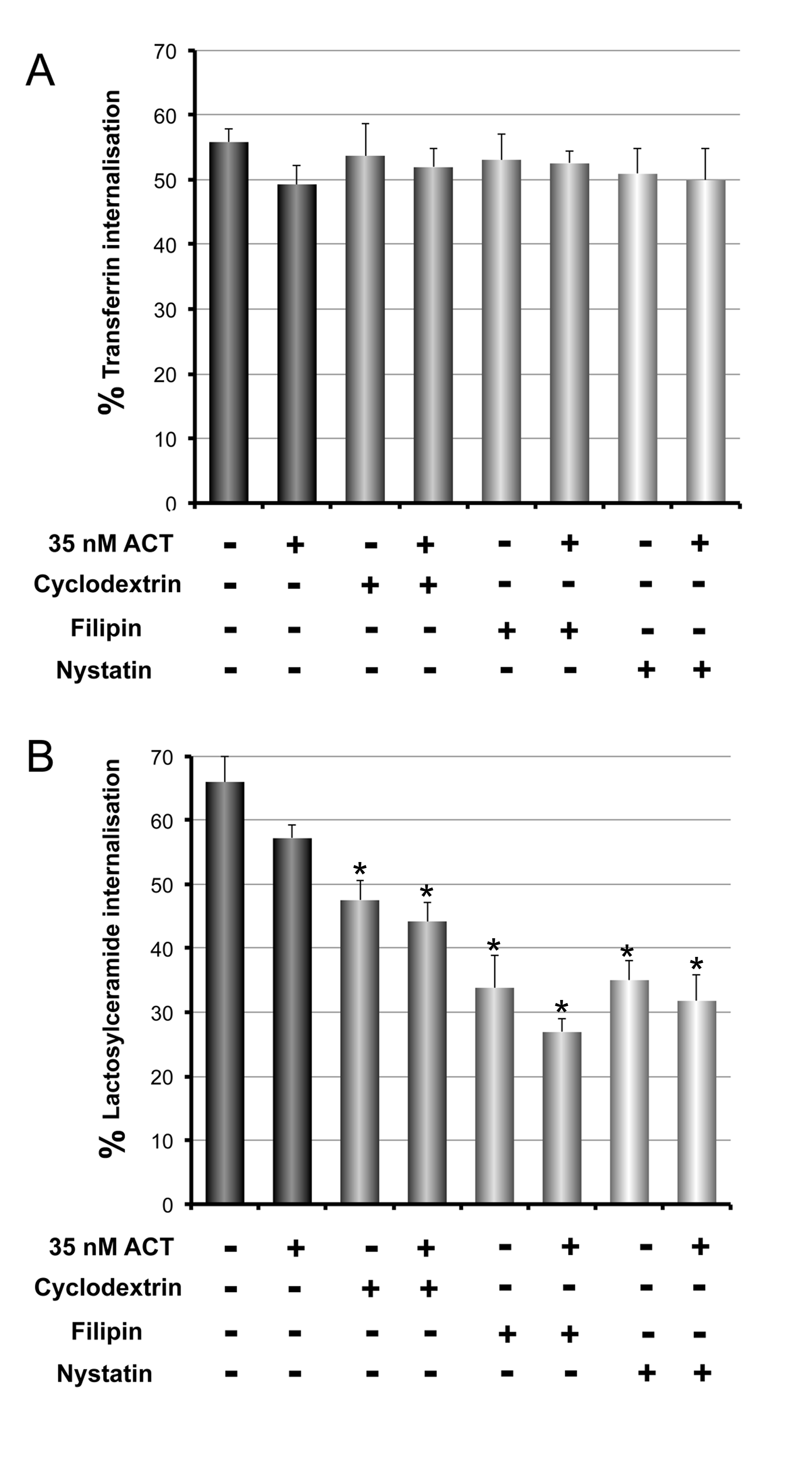

Supplement: Figure S2 — Effect of cholesterol-depletion on the internalisation of transferrin and BODIPY-LacCer by CHO-K1 cells. CHO-K1 cells were pre-incubated for 30 min at 37°C with three different cholesterol depleting agents, methyl-β-cyclodextrin (10 mM), nystatin (25 µg/ml) and filipin (3 µg/ml). (A) Internalisation of FITC-transferrin with or without cholesterol depleting agents. (B) Internalisation of BODIPY-lactosylceramide with or without cholesterol depleting agents. Internalisation was assessed by FACS as described in Materials and Methods. Data shown are the mean ± SD of at least three independent experiments, with *p<0.001 respect to non treated cells. (TIF) [file pone.0074248.s002.tif]

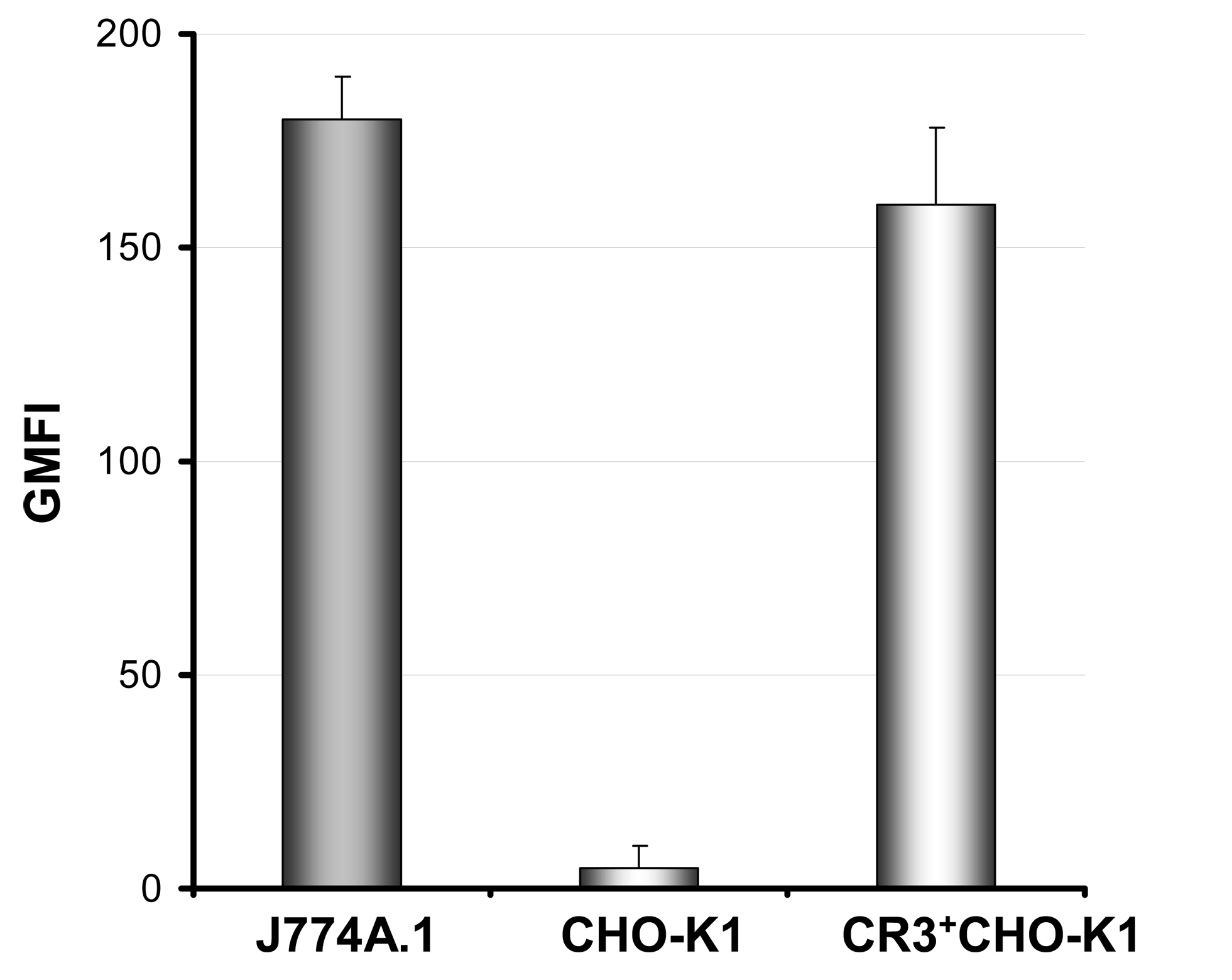

Supplement: Figure S3 — Control of effectiveness of CR3 transfection in CHO-K1 cells. Surface expression of the CR3 integrin was detected by flow cytometry in control CHO-K1 cells, in the transfected CR3+CHO-K1 cells and in the naturally CR3 expressing J774A.1 macrophages. Geometric mean fluorescence intensity (GMFI) was used to show CR3 expression. Data shown are the mean ± SD of at least three independent experiments. (TIF) [file pone.0074248.s003.tif]

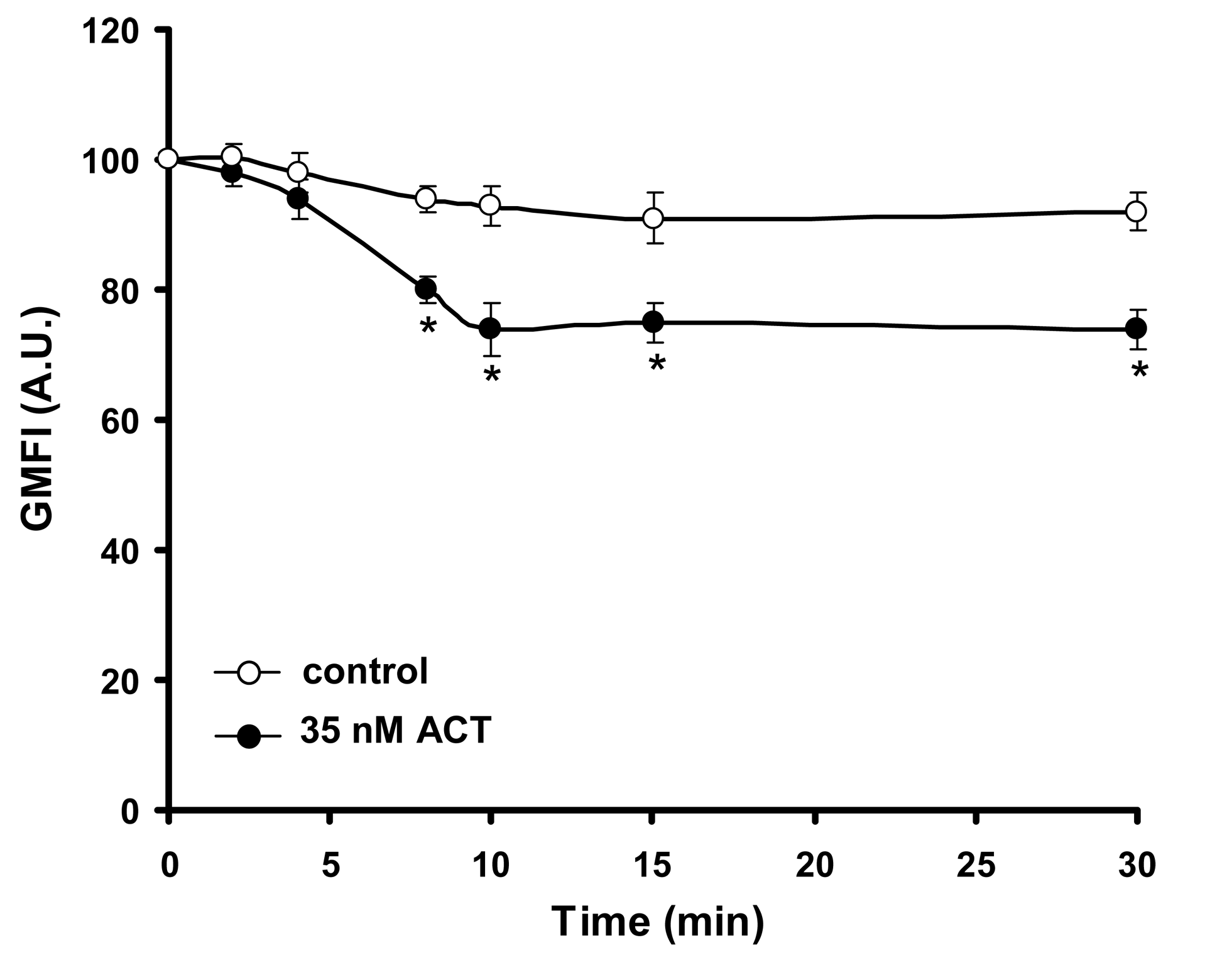

Supplement: Figure S4 — Time-course of ACT internalisation in CR3−J774A.1 cells. Addition of ACT (35 nM) to CR3−J774A.1 cells results in time-dependent internalisation of the toxin, as determined by flow cytometry and described in Materials and Methods. Geometric mean fluorescence intensities (GMFI) were used to show ACT internalisation. The data shown are the mean ± SD of at least three independent experiments, with ***p<0.001. (TIF) [file pone.0074248.s004.tif]

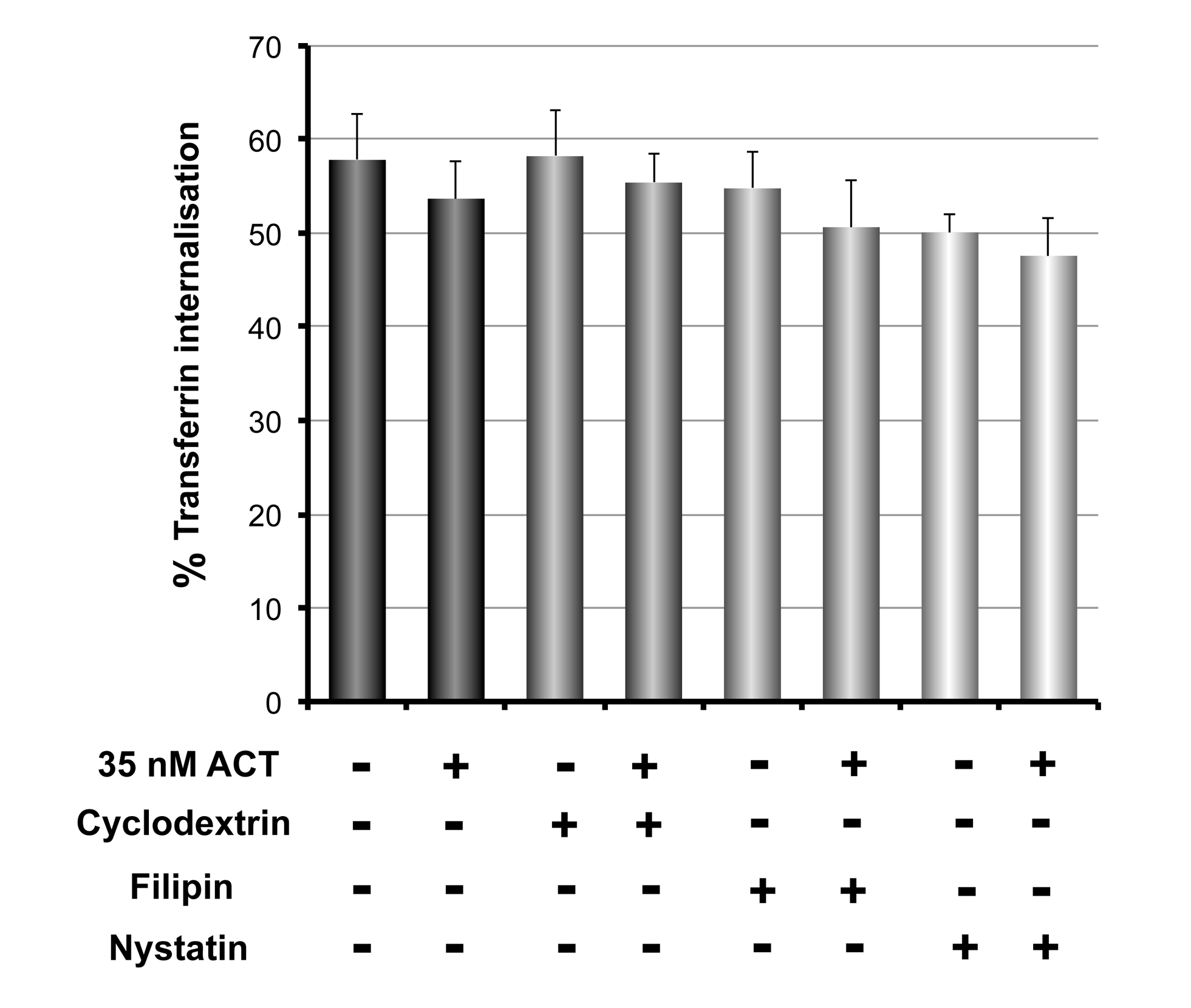

Supplement: Figure S5 — Effect of cholesterol-depletion on the internalisation of FITC-Transferrin by CR3−J774A.1 cells. CR3−J774A.1 cells were pre-incubated for 30 min at 37°C with three different cholesterol depleting agents, methyl-β-cyclodextrin (10 mM), nystatin (25 µg/ml) and filipin (3 µg/ml). Internalisation of FITC-transferrin with or without cholesterol depleting agents was assessed by FACS as described in Materials and Methods. Data shown are the mean ± SD of at least three independent experiments. (TIF) [file pone.0074248.s005.tif]

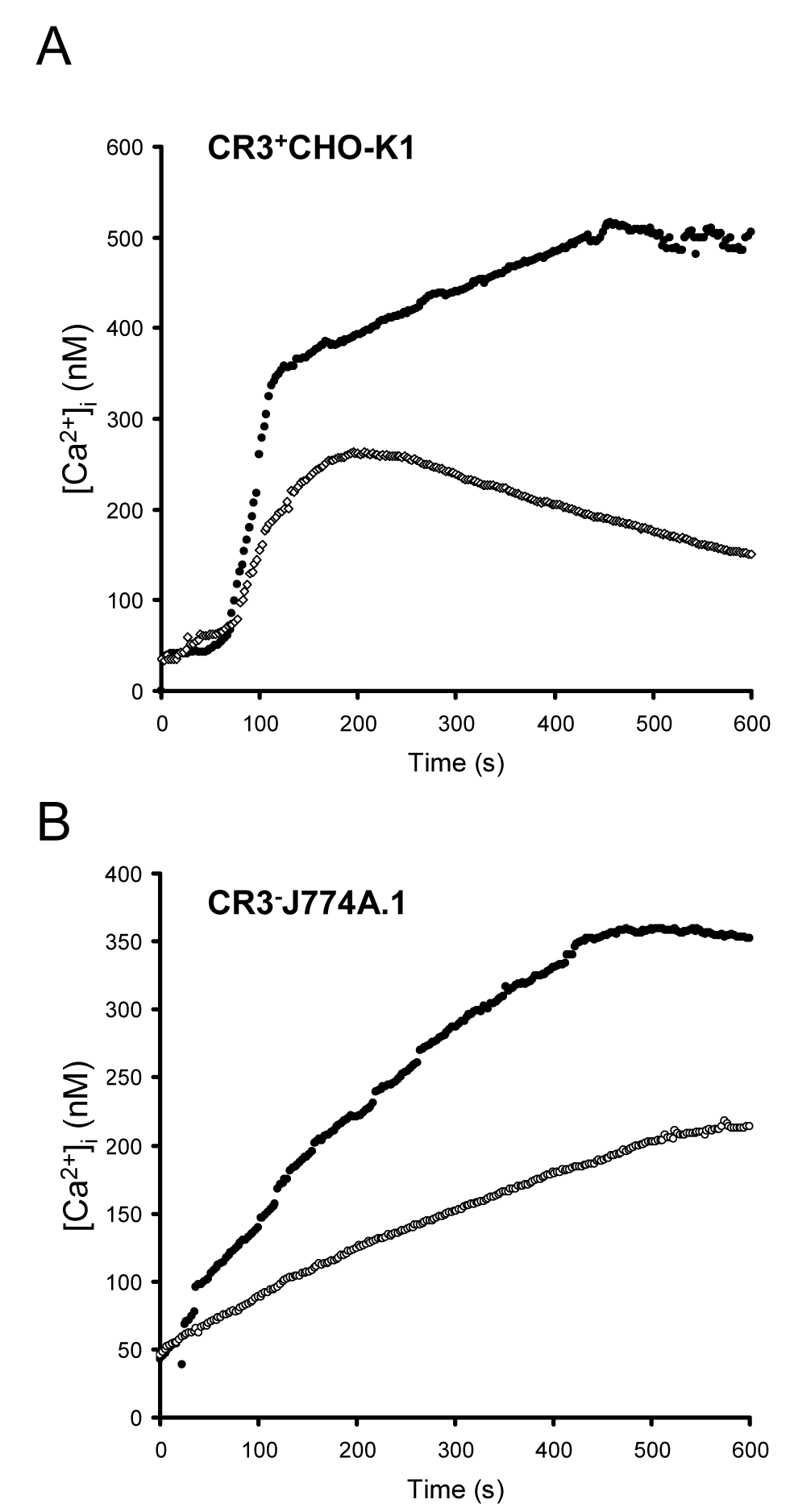

Supplement: Figure S6 — Kinetics of calcium influx as determined by FURA-2 fluorescence in CR3−J774A.1 and CR3+CHO-K1 cells. Calcium influx into (A) CR3+CHO-K1 and (B) CR3−J774A.1 cells was measured as previously described [18]. Briefly, cells grown on glass coverslips, were loaded with 2 µM fura2-AM for 30–45 min. The coverslips were mounted on a termostatized perfusion chamber on a Nikon Eclipse TE 300 based micro-spectrofluorometer and visualized with a ×40 oil-immersion fluorescence objective lens. At the indicated time, 35 nM ACT was added and the intracellular Ca2+ levels were determined using the method of Grynkiewicz et al. [31]. The ratio of light exited at 340 nm to that at 380 was determined with a Delta-Ram system (Photon Technologies International, Princeton). When required, cells were pre-incubated for 30 min with nifedipine, and then ACT was added. Data shown are the mean ± SD of three independent experiments. (TIF) [file pone.0074248.s006.tif]
